# SURF: A Self‐Supervised Deep Learning Method for Reference‐Free Deconvolution in Spatial Transcriptomics

**DOI:** 10.1002/advs.202505456

**Published:** 2025-08-26

**Authors:** Shuyu Liang, Zixia Zhou, Peng Huang, Junhu Fu, Jing Jiao, Yunxia Huang, Shichong Zhou, Guanlin Wang, Yuanyuan Wang, Yi Guo

**Affiliations:** ^1^ School of Information Science and Technology Fudan University Shanghai 200433 China; ^2^ Department of Radiation Oncology Stanford University Stanford CA 94305 USA; ^3^ Department of Ultrasound Fudan University Shanghai Cancer Center Shanghai 200032 China; ^4^ Department of Oncology Shanghai Medical College Fudan University Shanghai 200032 China; ^5^ Shanghai Key Laboratory of Metabolic Remodeling and Health Institute of Metabolism and Integrative Biology Centre for Evolutionary Biology Fudan University Shanghai 200032 China; ^6^ Shanghai Qi Zhi Institute Shanghai 200032 China; ^7^ Key Laboratory of Medical Imaging Computing and Computer Assisted Intervention of Shanghai Shanghai 200032 China

**Keywords:** deconvolution, deep learning, reference‐free, self‐supervised, spatial transcriptomics

## Abstract

Spatial transcriptomics has revolutionized tissue biology by enabling spatially resolved gene expression profiling. Nonetheless, current spot‐level spatial transcriptomic technologies consolidate signals from multiple cells, complicating cellular‐level analysis. Moreover, matched single‐cell references required by reference‐based deconvolution methods are frequently unavailable. To overcome these limitations, **we present SURF**, a reference‐free deconvolution tool that integrates high‐dimensional gene data analysis with self‐supervised deep learning to effectively model nonlinear gene interactions and leverage spot relationships. Benchmarking on both synthetic and real datasets shows that SURF consistently outperforms existing reference‐free methods and exceeds reference‐based approaches when appropriate references are absent. Applications across datasets with varying resolutions, species, spatial patterns, and tissue states demonstrate SURF's robust capacity to precisely represent tissue microenvironments. Importantly, SURF successfully identifies clinically significant epithelial‐to‐mesenchymal transition states within tumor regions in a dataset of human colorectal liver metastasis, highlighting its utility in uncovering critical biological mechanisms relevant to disease progression.

## Graphical abstract

SURF is a robust reference‐free deconvolution tool that integrates high‐dimensional spatial transcriptomics gene expression analysis with self‐supervised deep learning, enabling effective modeling of non‐linear gene interactions and spot relationships. SURF excels at delineating tissue architectures and significantly enhances high‐resolution analysis of spatial transcriptomics data, thereby supporting advanced biological discovery and innovative therapeutic research.

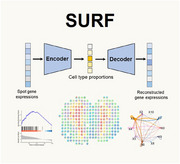

## 1 Introduction

Spatial transcriptomics (ST) has revolutionized our understanding of tissue organization by enabling the measurement of spatially resolved gene expressions.^[^ ^1^, ^2^, ^3^ ^]^ ST technologies can be categorized into two primary categories. Sub‐cellular resolution technologies, including MERFISH,^[^ ^4^ ^]^ SeqFISH+,^[^ ^5^ ^]^ Xenium,^[^ ^6^ ^]^ Stereo‐seq,^[^ ^7^ ^]^ and CosMx,^[^ ^8^ ^]^ facilitate detailed profiling of gene expression at high resolution, capturing subcellular information within tissues. Nonetheless, these methodologies frequently encounter specific limitations, including a limited number of detected genes, high expenses, restricted accessibility, substantial computational demands, and difficulties in accommodating large sample sizes. At the same time, multi‐cellular resolution technologies, including Slide‐seq,^[^ ^9^ ^]^ Visium,^[^ ^10^ ^]^ and ST,^[^ ^11^ ^]^ offer comprehensive whole‐transcriptome gene expression data that elucidate tissue architectures, making them especially advantageous for early‐stage discovery, hypothesis generation, and large‐scale clinical studies. However, these multi‐cellular resolution technologies often aggregate signals from multiple cells within each spot, complicating cell‐type‐specific analyses and hindering crucial biological signal explorations within the tissue microenvironments.

To address the challenge in analyzing and interpreting multi‐cellular resolution in ST data, computational algorithms to decompose the aggregated signals at each spot into cell‐type‐specific proportions have been developed, which can be broadly categorized into reference‐based and reference‐free deconvolution methods. Reference‐based methods, including SONAR,^[^ ^12^ ^]^ Cell2location,^[^ ^13^ ^]^ Redeconve,^[^ ^14^ ^]^ DestVI,^[^ ^15^ ^]^ DSTG,^[^ ^16^ ^]^ and GraphST,^[^ ^17^ ^]^ require matched single‐cell RNA sequencing (scRNA‐seq) datasets as references to assist the deconvolution process. However, acquiring matched scRNA‐seq references is consistently challenging due to sample availability, batch effects, and inter‐ and intra‐sample heterogeneities.

In contrast, reference‐free deconvolution methods have been developed to overcome the need for external references by extracting latent cell‐type factors directly from ST data. STdeconvolve employs latent Dirichlet allocation to identify latent cell type factors from gene expressions,^[^ ^18^ ^]^ whereas CFS utilizes independent component analysis to extract decomposition signals from multiple cell type mixtures.^[^ ^19^ ^]^ BayesTME builds on a generative Bayesian model to deconvolve spots into distinct cell types.^[^ ^20^ ^]^ The above three reference‐free methods ignore the nonlinear interactions among genes, which are essential for elucidating the complex biological mechanisms underlying gene expression. Moreover, STdeconvolve and CFS do not explicitly incorporate spot relationships. Spatially adjacent spots often share similar cell‐type compositions, reflecting close relationships, whereas spots with significant differences in gene expression typically represent distinct cellular environments, indicating distant relationships. BayesTME takes a step toward leveraging spot relationships by modeling neighboring spots as closely related, but it does not fully account for scenarios where adjacent spots may occasionally belong to distinct microenvironments. All these limitations highlight an opportunity for further refinement in reference‐free deconvolution methods.

Recent advancements in self‐supervised deep learning present exciting potential to address these challenges. Self‐supervised deep learning demonstrates strong performance in extracting meaningful representations from high‐dimensional nonlinear unlabeled data across computer vision tasks,^[^ ^21^, ^22^, ^23^ ^]^ making it particularly suitable for reference‐free deconvolution in ST data. Leveraging the advantages of self‐supervised deep learning, algorithms can adaptively model the nonlinear interactions among genes in ST data and flexibly integrate spot relationships, accurately capturing the underlying spatial structure and tissue heterogeneity.

Here, we developed SURF, an innovative **S**elf‐s**U**pervised deep learning **R**eference‐**F**ree method for spot‐level spatial transcriptomic deconvolution. To the best of our knowledge, SURF is the first self‐supervised deep learning tool designed for reference‐free deconvolution, presenting a novel algorithmic framework for addressing this challenge. It employs an autoencoder architecture to model nonlinear gene interactions, enabling the extraction of informative latent representations. SURF also leverages contrastive learning to assimilate spot relationships, incorporating spatial information and biological prior knowledge into the model. We benchmarked SURF against both reference‐free and reference‐based methods using synthetic datasets, demonstrating its superior performance in accurately recovering cell‐type compositions. Notably, SURF demonstrated exceptional performance in scenarios where appropriate scRNA‐seq references are lacking, a common challenge in real‐world applications. We also showed SURF better characterized tissue structures and resolved cellular compartments compared to other reference‐free methods in real‐world datasets, including the mouse main olfactory bulb, mouse spermatogenesis, and human dorsolateral prefrontal cortex. Furthermore, SURF successfully identified three epithelial‐to‐mesenchymal transition (EMT) states within the tumor region in the human colorectal liver metastasis dataset. Overall, SURF exhibits more robust performance in delineating tissue architectures and facilitating high‐resolution biological analyses in spot‐level ST datasets. This tool lays a solid foundation for uncovering biologically meaningful insights, advancing mechanistic investigations, and driving therapeutic discovery.

## 2 Results

### 2.1 Workflow of SURF

**Figure 1:**
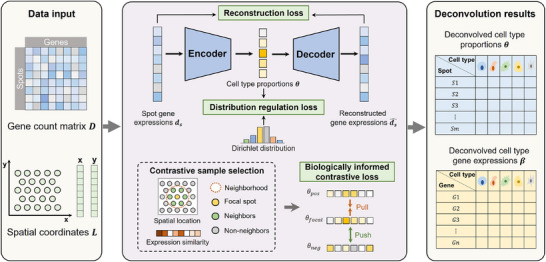
**Overview of SURF**. SURF requires the gene count matrix and spatial coordinates of spatial transcriptomic data as input (left), and outputs deconvolved cell type proportions of each spot and deconvolved cell type gene expressions of each cell type (right). SURF employs an autoencoder architecture to perform deconvolution, accounting for both reconstruction loss and distribution regulation loss. Moreover, SURF uses the biologically informed contrastive loss to include spot relationships in the model.

SURF employs self‐supervised deep learning to deconvolve ST data in a reference‐free manner, presenting a powerful tool for high‐resolution tissue characterization (**Figure** **1**). Taking the gene count matrix ***D*** and spatial coordinates ***L*** of spots as input, SURF predicts the deconvolved cell type proportions (CTPs) ***θ*** and the deconvolved cell type transcriptional profiles ***β***. For each spot, the encoder extracts informative feature representation from the input gene expression vector ***d***_***s***_, generating the cell type proportion prediction ***θ*** = Softmax(MLP(***d***_***s***_)). The multilayer perceptron (MLP) layers of the encoder capture the complicated nonlinear interactions among genes. Subsequently, ***θ*** is fed into the decoder to reconstruct the gene expressions $\hat{d_{s}}=\mathrm{Softmax}\left(\beta \theta +b_{0}\right)$, where ***b***_0_ represents a *G*‐dimensional bias vector of the linear layer. A reconstruction loss *L_recon_* guarantees that the network captures maximal effective information from the high‐dimensional gene expression input. SURF introduces a distribution regulation loss *L*_*distri* − *reg*_ that constrains the predicted CTPs to obey a Dirichlet distribution, taking into account the statistical characteristics of CTPs. The biologically informed contrastive loss *L_contras_* incorporates the biological knowledge of spot relationships into the model. Spatially adjacent spots with high gene expression similarities are likely to possess similar cell type compositions, reflecting close relationships, whereas spots with significant gene expression disparities tend to exhibit distinct cellular constituents, indicating distant relationships.^[^ ^24^, ^25^, ^26^ ^]^ Using contrastive learning, SURF pulls spots with close relationships closer, leading to similar cell type composition predictions. Conversely, SURF pushes spots with distant relationships further apart, promoting more dispersion and diversity in cell type proportion predictions.

### 2.2 Benchmarking Simulated ST Data With Different Spatial Patterns

**Figure 2:**
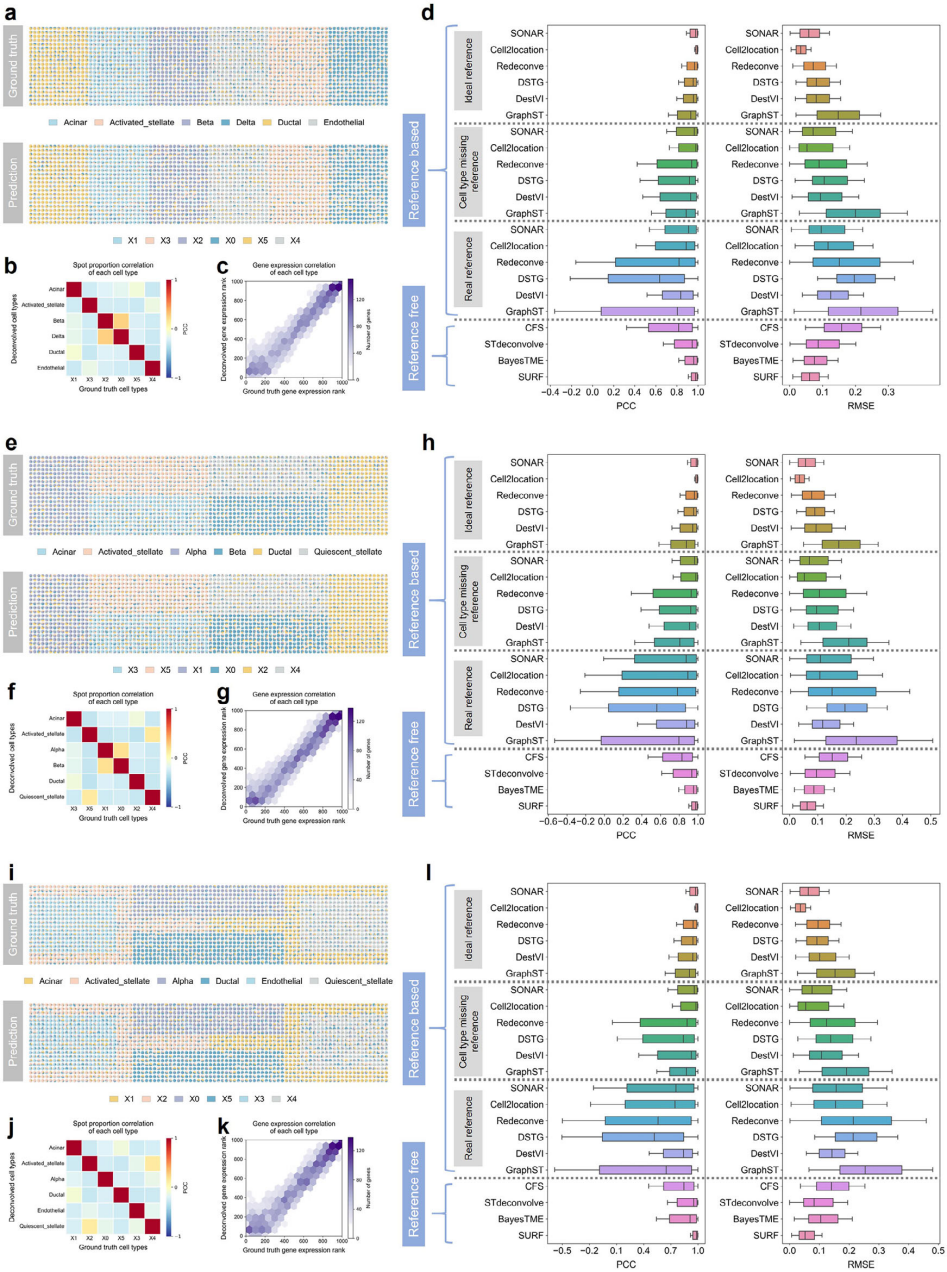
**Benchmarking in simulated spatial transcriptomics data with layer pattern** a–d), block pattern e–h), and background pattern i–l). a,e,i) Ground truth (top) and SURF predictions (bottom) of cell type proportions in different spatial patterns. The pie chart at each spot represents the cell type proportions. b,f,j) Pearson correlations between the deconvolved and ground truth cell type proportions of different cell types. c,g,k) Gene expression rankings in the deconvolved cell type transcriptional profiles compared to gene expression rankings in the ground truth cell type transcriptional profiles. The color of the hexagon represents the number of genes located in this region (only hexagons with 5 or more genes are shown). d,h,l) Boxplots of Pearson correlation coefficient (PCC) and root mean square error (RMSE) of different deconvolution methods. Center line, median value; box limits, upper and lower quartiles; whiskers, 0.5× interquartile range. All the methods are divided into two categories: reference‐based and reference‐free. Reference‐based methods using ideal reference (top), cell type missing reference (middle), and real reference (bottom) are shown, respectively.

To evaluate the performance of SURF, we used scRNA‐seq to simulate ST data to model different tissue spatial patterns. The simulations were based on a well‐annotated human pancreas scRNA‐seq dataset.^[^ ^27^ ^]^ To mimic the fact that different cell types often concentrate in specific subregions, we randomly selected six cell types, each predominantly occupying a distinct subregion. We then simulated three spatial patterns to illustrate typical region distributions: layer pattern, block pattern, and background pattern (**Figure** **2**a,e,i; Figure S1, Supporting Information). In each simulation, scRNA‐seq data were aggregated to generate 20 × 90 spots.

SURF successfully recovered cell type distributions across all three spatial patterns, precisely delineating distinct subregions (Figure 2a,e,i). The predicted CTPs matched well with the ground‐truth CTPs (Figure 2b,f,j), and the deconvolved cell type transcriptional profiles showed strong consistency with the true expression profiles (Figure 2c,g,k). We compared SURF with reference‐based methods, including SONAR, Cell2location, Redeconve, DestVI, DSTG, and GraphST (Figure 2d,h,l), across three reference conditions: ideal reference, cell type missing reference, and real reference. The ideal reference was the original scRNA‐seq data used to generate the simulated ST data. As expected, all the reference‐based methods exhibited robust performance with the ideal reference, perfectly aligning with the cell type composition. However, in real‐world applications, such ideal references are rarely available. We generated the “cell type missing” reference by excluding the acinar cell type and designated the “real reference” from a different scRNA‐seq sample in the pancreas study.^[^ ^27^ ^]^ The performance declined markedly across all reference‐based methods in these two scenarios with unmatched references. Notably, SURF consistently performed well in all three reference conditions, as reference‐free methods are independent of scRNA‐seq reference. In comparison with other reference‐free methods, including CFS, STdeconvolve, and BayesTME, SURF consistently achieved higher Pearson correlation coefficients (PCC) and lower root mean square errors (RMSE) across spots in all three spatial patterns, demonstrating its accuracy in recovering cell types (Figure 2d,h,l).

To evaluate performance under more biologically realistic conditions, we further simulated another set of ST data to reflect more complex scenarios. In real biological tissues, certain cell types tend to localize within specific subregions, while others are more diffusely distributed. To imitate this, we randomly selected five cell types as dominant cell types, each enriched within a specific subregion. And we used three other cell types that were diffusely distributed throughout the region. We simulated three kinds of spatial patterns (Figures S2 and S3a,e, i, Supporting Information). In all spatial patterns, we aggregated single‐cell data to generate 20 × 75 spots. Consistent results were observed across all patterns (Figure S3, Supporting Information). Reference‐based methods performed well when an ideal reference was available, but exhibited substantial performance drops when using incomplete or real references. In contrast, SURF consistently outperformed reference‐based approaches under non‐ideal reference conditions across all three spatial patterns. Compared to other reference‐free deconvolution methods, SURF better recovered the cell type proportions, achieving the highest median PCC and the lowest median RMSE across all the spots in different spatial patterns. These results further demonstrate that SURF is robust and accurate across a variety of spatial configurations.

### 2.3 SURF Revealed the Fine‐Scale Structure of the Rostral Migratory Stream in the Mouse Main Olfactory Bulb

**Figure 3:**
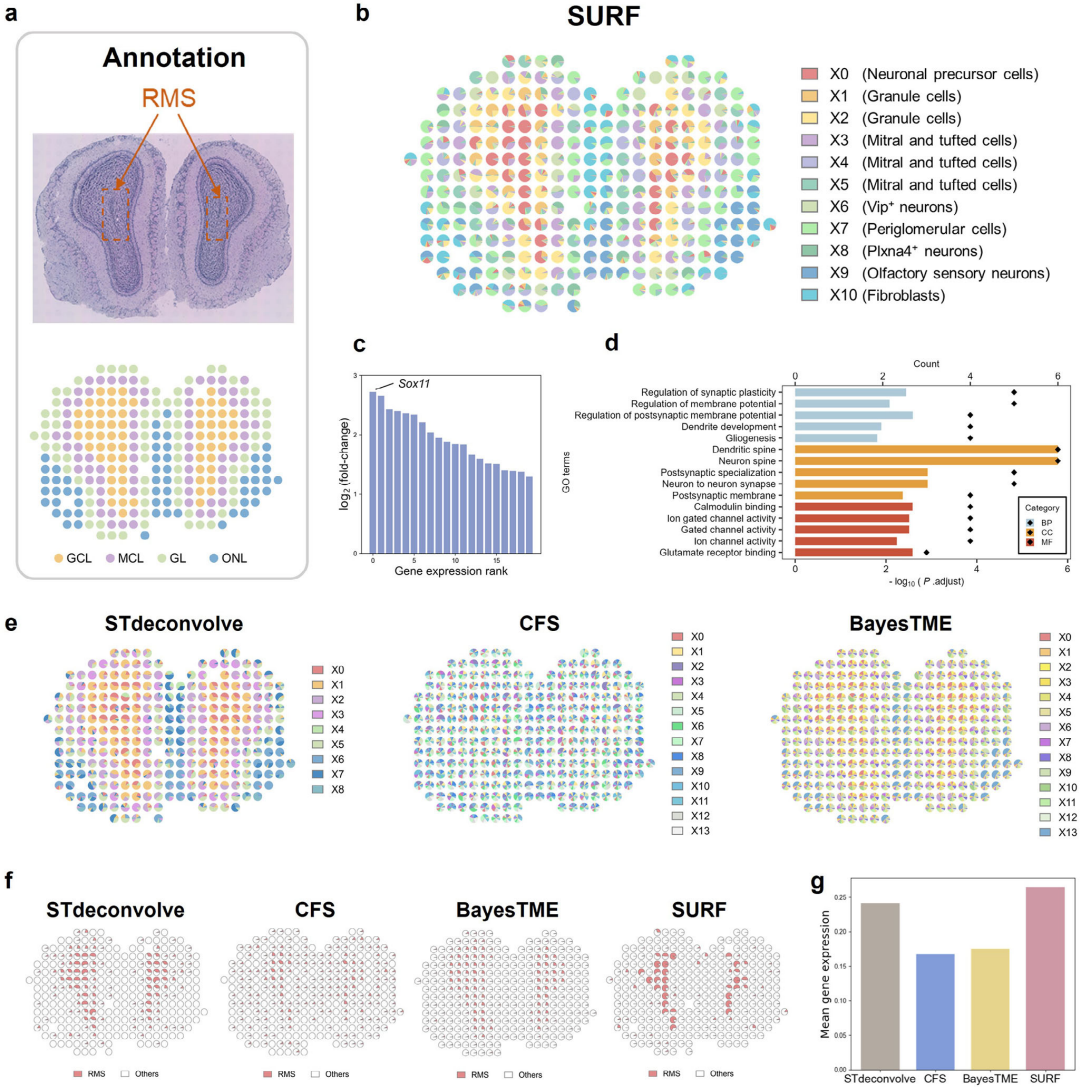
**Deconvolution results of the mouse main olfactory bulb dataset**. a) H&E staining image and spot‐level annotations of the mouse main olfactory bulb dataset.^[^ ^11^ ^]^ The red box indicates the area where the rostral migratory stream (RMS) is located. b) Deconvolution results of SURF. The pie chart at each spot represents the cell type proportions of this spot. c) Top twenty upregulated genes of cell type X0 (neuronal precursor cells) in SURF's prediction. d) Gene ontology enrichment analysis of upregulated genes in cell type X0 (neuronal precursor cells). GO ontology enrichment analysis was performed using a one‐sided hypergeometric test. *P* values were adjusted using the Benjamini–Hochberg method. e) Deconvolution results of other reference‐free methods. The pie chart at each spot represents the cell type proportions of this spot. f) Distributions of the RMS‐related cell type identified by different methods. g) Mean expression of marker genes *Sox11* in the RMS‐related cell type of different methods.

We further applied SURF to several real ST datasets to demonstrate its capability in recovering the cell type proportions in complex tissues. The first dataset was the mouse main olfactory bulb (MOB) data at a resolution of 100 µm.^[^ ^11^ ^]^ The MOB is a crucial brain region responsible for processing olfactory information and is characterized by specific anatomic layers.^[^ ^28^ ^]^ This dataset was annotated at the spot level in a previous study (**Figure** **3**a).^[^ ^29^ ^]^ The rostral migratory stream (RMS), a fine structure, is localized within the inner layer of MOB. SURF successfully identified eleven cell types within the MOB tissue (Figures 3b and S4a, Supporting Information), precisely delineating the layered structure of the MOB. Notably, SURF identified a specific cell type, X0 (neuronal precursor cells), localized within the granule cell layer, corresponding to the RMS region. To further validate the correlation between the cell type X0 (neuronal precursor cells) and the RMS, we analyzed the upregulated genes in the deconvolved transcriptional profile in X0 (Figure 3c; Table S1, Supporting Information) and identified high expression of *Sox11*, *Mag*, *Tubb2b*, etc. *Sox11*, in particular, has been reported as a marker gene for RMS,^[^ ^28^ ^]^ indicating that X0 (neuronal precursor cells) likely represents a cell type associated with the RMS. Moreover, we characterized the X0 cell type by conducting gene ontology (GO) enrichment analysis (Figure 3d) using its upregulated genes. The “regulation of synaptic plasticity” term highlights the dynamic nature of neuronal precursor cells in modulating synaptic plasticity, a process critical for neuronal integration.^[^ ^30^ ^]^ The enrichment of the terms “neuron to neuron synapse” and “ion channel activity” underscores the role of the X0 cell type (neuronal precursor cells) in synapse formation and ion transport, both of which are essential for the development of neural networks.^[^ ^31^, ^32^ ^]^

We compared the deconvolution results of SURF with other reference‐free methods, STdeconvolve, CFS, and BayesTME. STdeconvolve identified nine cell types (Figure 3e, left; Figure S4b, Supporting Information) and recovered the primary structure of the MOB, including the RMS‐associated cell type X0 (Figure 3f). High expression of *Sox11* was also observed in X0 of STdeconvolve (Figures S4c and Table S2, Supporting Information). CFS deconvolved the MOB data into fourteen cell types, but did not elucidate the layered structure as distinctly as SURF (Figure 3e, middle**;** Figure S4d, Supporting Information). A cell type X0 associated with RMS was also identified by CFS with high expression of *Sox11* (Figure 3f; Figure S4e and Table S3, Supporting Information). BayesTME identified fourteen cell types (Figure 3e, right; Figure S4f, Supporting Information) and similarly resolved the layered structure of the MOB, including an RMS‐related cell type X0 exhibiting *Sox11* expression (Figure 3f; Figure S4g and Table S4, Supporting Information). To quantitatively evaluate the deconvolution accuracy for the RMS‐related cell type, we calculated the mean expression of the RMS‐related marker gene *Sox11* in the deconvolved RMS‐related cell type across all reference‐free methods (Figure 3g). Significantly higher expression of *Sox11* in SURF's deconvolution results compared to other methods indicates its enhanced efficacy in characterizing fine‐scale structures.

To further evaluate SURF's performance, we benchmarked several reference‐based deconvolution methods mentioned using a well‐annotated MOB single‐cell dataset from Tepe et al. as the reference.^[^ ^28^ ^]^ Most reference‐based methods, including SONAR, cell2location, GraphST, Redeconve, and DestVI, were able to accurately recover the multi‐layered architecture of the MOB, similar to SURF. In contrast, DSTG was less effective and showed reduced accuracy, displaying noticeable misclassification across different layers (Figures S5b, S6, Supporting Information). Notably, the “immature” cell type, which specifically marks the fine‐scale RMS structure, was accurately mapped to the inner MOB layer by SONAR, cell2location, and Redeconve. Although GraphST, DestVI, and DSTG also detected “immature” cells in the RMS region, they incorrectly assigned some of them to other non‐RMS areas as well (Figure S5c, Supporting Information). To further compare each method's ability to identify the RMS structure, we calculated the mean expression of the RMS marker gene *Sox11* in the deconvolved “immature” population (Figure S5d, Supporting Information). SURF achieved the highest mean *Sox11* expression, outperforming all reference‐based methods in accurately delineating the RMS structure.

### 2.4 SURF Resolved the Cellular Compartments of Mouse Spermatogenesis

**Figure 4:**
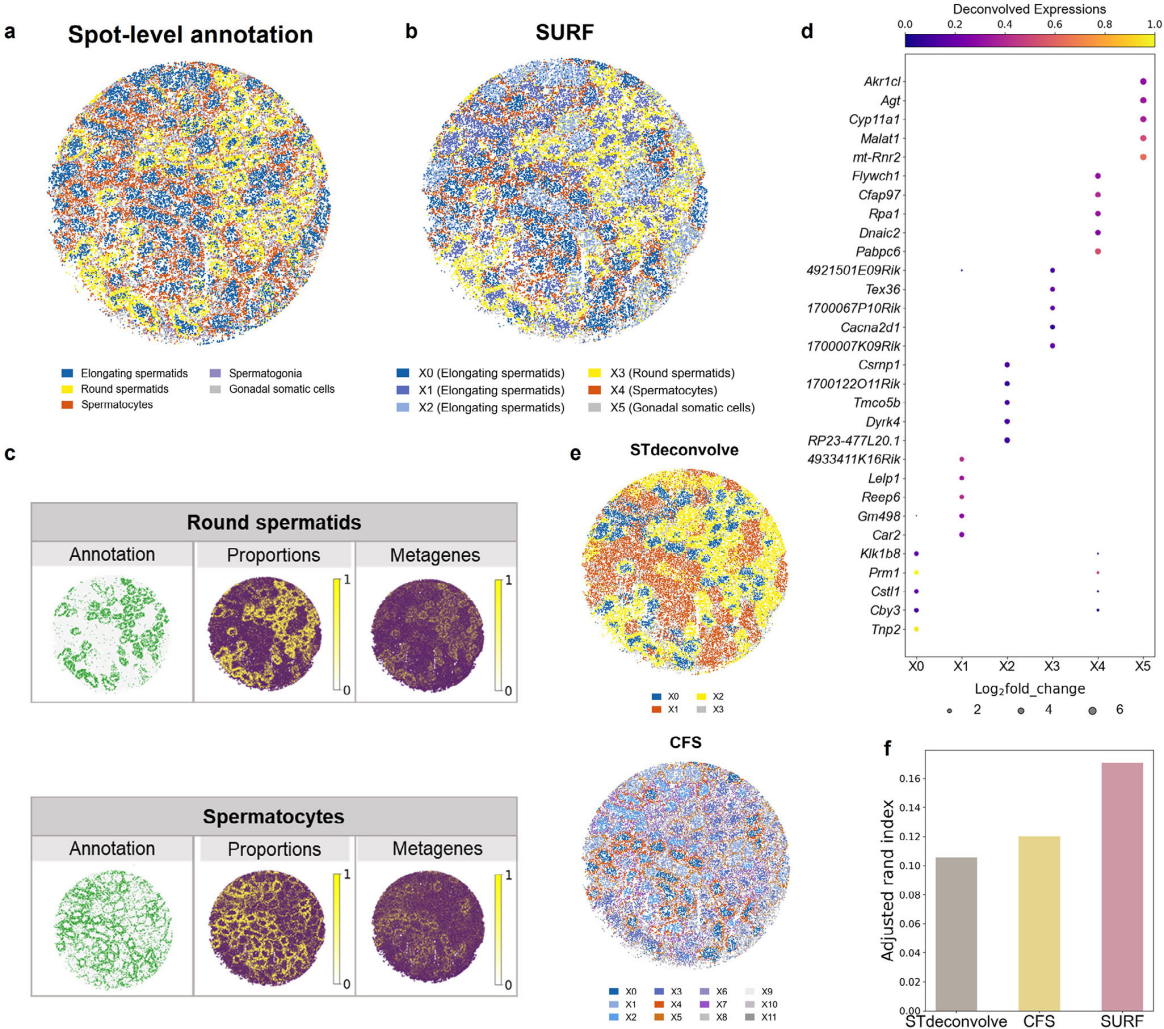
**Deconvolution results of the mouse spermatogenesis dataset**. a) Spot‐level cell type annotation. b) Deconvolution results of SURF. The color at each spot represents the dominant cell type of this spot. c) Spot‐level annotation, cell type proportion predictions, and metagenes expressions of two cell types from SURF's deconvolution. The metagene expressions are defined as the mean of the top ten upregulated genes for each cell type. d) Top five upregulated genes of each SURF's deconvolved cell types. The size of the circle represents the $\log_{2}\mathrm{fold}\_\mathrm{change}$ value of each gene (values less than zero were set to zero), and the color of the circle represents the deconvolved expressions of each gene. e) Deconvolution results of STdeconvolve and CFS (BayesTME were unable to perform on this dataset due to high memory usage). The color at each spot represents the dominant cell type of this spot. f) Adjusted rand index between dominant cell type predictions and spot‐level annotations of three reference‐free methods.

To evaluate SURF's performance across different ST platforms, we further applied SURF to a mouse spermatogenesis dataset measured using Slide‐seq at a resolution of 10 µm. ^[^ ^33^ ^]^ Spermatogenesis, the biological process of sperm production, occurs within spatially confined functional units termed seminiferous tubules.^[^ ^33^ ^]^ This dataset was annotated into five primary cell types at the spot level according to the original study (**Figure** **4**a; Figure S7a, Supporting Information), with the regions indicating small, irregular, round or oval structures corresponding to the seminiferous tubules. Notably, elongating spermatids were predominantly located at the center of the seminiferous tubules, forming a typical “background pattern” as shown in our simulated datasets.

SURF deconvolved the data into six cell types (Figure 4b; Figure S7b, Supporting Information), accurately capturing the spatial organization of seminiferous tubules. SURF effectively distinguished round spermatids and spermatocytes cell types, precisely assigning them around elongating spermatids cells. The deconvolved cell type proportions of these two cell types exhibited high concordance with the spot‐level annotations (Figure 4c). The metagenes predicted by SURF provided supplementary evidence, reinforcing the reliability of these predictions. The illustrated top five upregulated genes of each cell type provided enhanced interpretability for the deconvolution results (Figure 4d; Table S5, Supporting Information). For instance, *Prm1* and *Tnp2* in X0 (elongating spermatids) are two reported marker genes for the elongating spermatids cell type, which are essential for germ cell development and spermiogenesis.^[^ ^34^ ^]^

We also performed STdeconvolve and CFS on this dataset. STdeconvolve deconvolved four cell types in the dataset (Figure 4e, top; Figure S7c and Table S6, Supporting Information). Although STdeconvolve partially resolved the structure of the spermatogenesis dataset, it struggled to identify round spermatids and spermatocytes precisely. The deconvolved distributions of these two cell types were confused with elongating spermatids. While CFS offered improved deconvolution compared to STdeconvolve, it still fell short in accurately identifying round spermatids and spermatocytes compared to SURF. (Figure 4e, bottom; Figure S7d and Table S7, Supporting Information). We calculated the adjusted rand index (ARI) between the spot‐level dominant cell type predictions (determined by the cell type with the highest CTP in each spot) and the annotations (Figure 4f). Notably, SURF outperformed STdeconvolve and CFS with the highest ARI.

We also applied reference‐based deconvolution methods using a mouse germ cell single‐cell dataset to assess their ability to resolve the spatial architecture of seminiferous tubules.^[^ ^35^ ^]^ Methods such as SONAR, cell2location, GraphST, and Redeconve accurately captured the spatial organization of the seminiferous tubules, showing strong concordance with spot‐level annotations. In contrast, DestVI and DSTG demonstrated reduced accuracy, with frequent misclassification of major germ cell populations such as elongating spermatids, round spermatids, and spermatocytes (Figures S8b, S9, Supporting Information). We calculated the ARI between the dominant cell type predictions for each spot and the spot‐level annotations (Figure S8c, Supporting Information). SURF maintained its superior performance on this dataset, achieving a higher ARI than most reference‐based methods and performing comparably to GraphST.

### 2.5 SURF Characterized the Multi‐Layer Structure in the Human Dorsolateral Prefrontal Cortex

**Figure 5:**
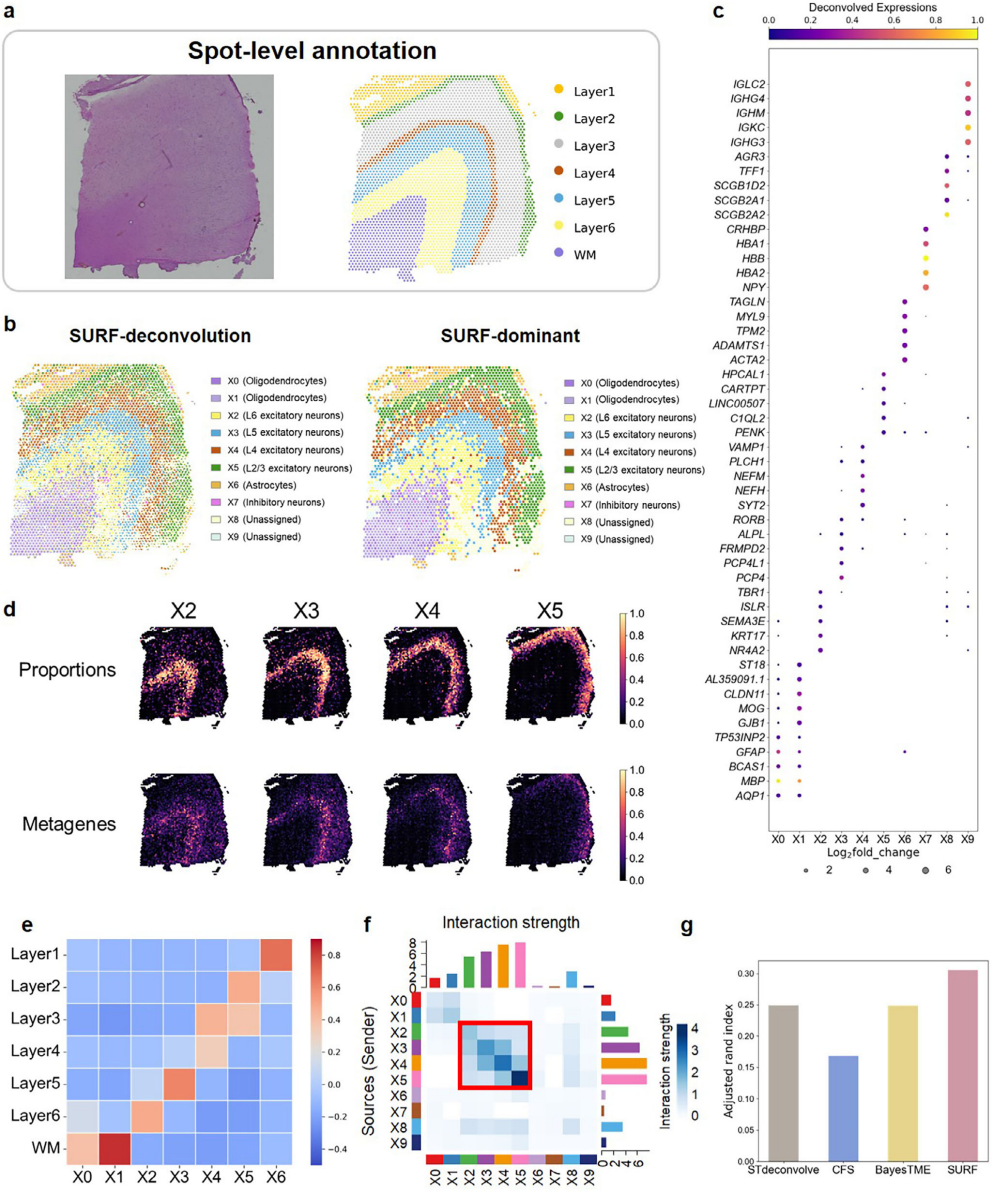
**Deconvolution results of the human dorsolateral prefrontal cortex dataset**. a) H&E staining image and spot‐level annotations of the human dorsolateral prefrontal cortex.^[^ ^36^ ^]^ b) Deconvolution results of SURF. Left: deconvolved cell type proportions. The pie chart at each spot represents the cell type proportions of this spot. Right: dominant cell types. The color at each spot represents the dominant cell type of this spot. c). The top five upregulated genes of each SURF's deconvolved cell types. The size of the circle represents the $\log_{2}\mathrm{fold}\_\mathrm{change}$ value of each gene (values less than zero were set to zero), and the color of the circle represents the deconvolved expressions of each gene. d) Visualizations for four SURF's deconvolved cell type proportions and their corresponding metagenes. The metagene expressions are defined as the mean of the top ten upregulated genes for each cell type. e) Spatial correlation between the deconvolved cell types of SURF and the spot‐level annotated domains. f) Plot of spatially resolved interaction strength among different deconvolved cell types of SURF. The thickness of the line indicates the strength of the interaction. g) Adjusted rand index between dominant cell type predictions and spot‐level annotations of different reference‐free methods.

To broaden the applicability of SURF to human tissues, we analyzed a human dorsolateral prefrontal cortex (DLPFC) dataset using Visium technology at a resolution of 55 µm.^[^ ^36^ ^]^ The DLPFC, vital for working memory, cognitive flexibility, and abstract reasoning,^[^ ^37^ ^]^ presents a typical “layer pattern” structure comprising six layers of dorsolateral prefrontal cortex and white matter (WM) (**Figure** **5**a). SURF identified ten deconvolved cell types (Figure 5b; S10a, Supporting Information), which corresponded well with the spot‐level annotation. Specifically, cell types X0 (oligodendrocytes) and X1 (oligodendrocytes) were consistent with the WM region, whereas X2 (L6 excitatory neurons), X3 (L5 excitatory neurons), X4 (L4 excitatory neurons), X5 (L2/3 excitatory neurons), and X6 (astrocytes) recapitulated the anticipated layer structure. We examined the biological relevance of the top five upregulated genes of each deconvolved cell type (Figure 5c; Table S8, Supporting Information). Among them, the upregulated gene *PCP4* of X3 (L5 excitatory neurons) is an L5 marker gene and is closely linked to brain activity.^[^ ^36^, ^38^ ^]^ The distribution of four representative neuronal cell types and the spatial expression patterns of their corresponding metagenes were visualized (Figure 5d). The spatial distribution inferred by SURF closely corresponded with the spatial expression patterns of the respective metagenes. The spatial correlation heatmap illustrated the alignment between deconvolved cell types and spot‐level annotation (Figure 5e), confirming the accuracy of SURF's prediction. We further analyzed the spatially resolved interaction strength in this dataset using CellChat,^[^ ^39^ ^]^ identifying strong cell‐cell communications between adjacent cortex layers (Figure 5f).

We compared SURF's performance with three other reference‐free methods, including STdeconvolve, BayesTME, and CFS (Figure S10b–d and Tables S9–S11, Supporting Information). All three comparative methods successfully identified WM regions and several cortical layers. However, specific layers, particularly Layers 3 to 6, were not consistently delineated and appeared mixed. We quantitatively calculated the ARI between the spot‐level dominant cell type predictions and the annotations. SURF achieved the highest ARI among all three methods, highlighting its effective performance in precisely characterizing multi‐layer tissue structures (Figure 5g).

For reference‐based deconvolution, we used a single‐nucleus dataset ^[^ ^40^ ^]^ from the human post‐mortem brain as the reference. Most reference‐based methods performed unsatisfactorily, failing to clearly resolve the different neural layers, except for GraphST, which showed clear layer distinction (Figures S11b, S12, Supporting Information). The ARI calculation further confirmed that SURF outperformed the majority of reference‐based methods and achieved performance comparable to GraphST (Figure S11c, Supporting Information).

### 2.6 SURF Identified Epithelial‐to‐Mesenchymal Transition Subtypes in Human Colorectal Liver Metastasis

**Figure 6:**
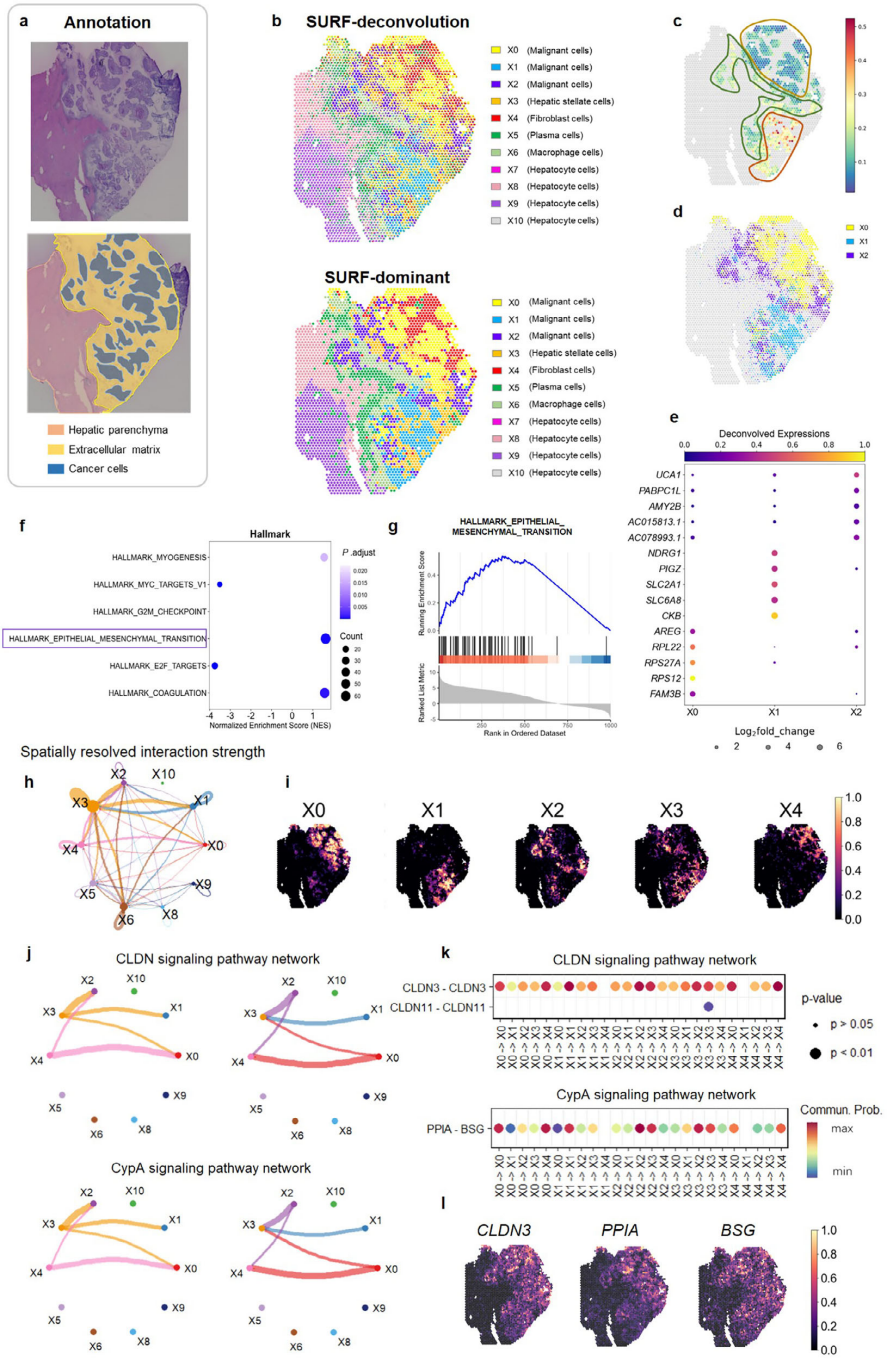
**Deconvolution results of the human colorectal liver metastasis dataset**. a) H&E staining image and annotations of the human colorectal liver metastasis dataset.^[^ ^41^ ^]^ b) Deconvolution results of SURF. Top: deconvolved cell type proportions. The pie chart at each spot represents the cell type proportions of this spot. Bottom: dominant cell types. The color at each spot represents the deconvolved dominant cell type of this spot. c) Epithelial‐to‐mesenchymal transition scores in the cancer area. d) Cell type distributions of three cancer‐related cell types deconvolved by SURF. e) Top five upregulated genes of the three SURF's deconvolved cell types in the cancer region. The size of the circle represents the $\log_{2}\mathrm{fold}\_\mathrm{change}$ value of each gene (values less than zero were set to zero), and the color of the circle represents the deconvolved expressions of each gene. f) Hallmark gene set enrichment analysis to explore the differences between SURF's cell type X0 and X1. Each dot represents a gene set. The horizontal axis represents the normalized enrichment score. The size of the dot represents the number of genes enriched in the gene set, and the color represents the *P*.adjust value. Statistical significance was assessed using a two‐sided permutation test. *P* values were adjusted using the Benjamini–Hochberg method. g)> Enrichment plot of the HALLMARK_EPITHELIAL_MESENCHYMAL_TRANSITION gene set. h) Plot of spatially resolved interaction strength among different deconvolved cell types of SURF. The thickness of the line indicates the strength of the interaction. i) Visualizations of SURF's deconvolved cell type proportions for five cell types, mainly located in the extracellular matrix (ECM) region or cancer region. j) Interactions between cell types in the cancer region and the ECM region within two key epithelial‐to‐mesenchymal transition‐related signaling pathway networks. Drawings on the left represent interactions from cancer cell types to ECM cell types, while drawings on the right depict interactions from ECM cell types to cancer cell types. k) Ligand‐receptor pairs associated with ECM‐related cell types (X3, X4) and cancer‐related cell types (X0, X1, and X2) in two pathways. l) Visualizations of the distributions of three important ligands or receptors.

To illustrate the applicability of SURF in disease tissues, we applied it to a human colorectal liver metastasis (CRLM) dataset generated using the Visium platform at a resolution of 55 µm (**Figure** **6**a).^[^ ^41^ ^]^ Liver metastasis is a primary contributor to mortality associated with colorectal cancer.^[^ ^42^ ^]^ The deconvolution of colorectal liver metastasis (CRLM) samples can provide insights into the tumor microenvironment for potential therapeutic targets. SURF deconvolved the sample into eleven distinct cell types (Figure 6b; Figure S13a and Table S12, Supporting Information), with X0 (malignant cells), X1 (malignant cells), and X2 (malignant cells) localized in cancerous regions, whereas X3 (hepatic stellate cells) and X4 (fibroblast cells) were spatially associated to the annotated extracellular matrix (ECM) regions. X5 (plasma cells) and X6 (macrophage cells) were located in the transition zone between tumor and normal regions, and X7‐X10 (hepatocyte cells) corresponded to the hepatic parenchyma. This dataset shows a “block pattern” with different cell types localized in separate block‐shaped regions.

EMT is a crucial process in cancer progression.^[^ ^43^ ^]^ Using EMT gene signatures from the EMTome database,^[^ ^44^ ^]^ we calculated EMT scores within the CRLM cancer regions (Figure 6c). Notably, the predicted malignant cell types X0, X1, and X2 corresponded to different EMT states: epithelial (X0, characterized by relatively lower EMT scores), mesenchymal (X1, characterized by relatively higher EMT scores), and intermediate state (X2, with EMT scores within a moderate range) (Figure 6c,d). We also illustrated the top five upregulated genes for these three cell types (Figure 6e). X0 (epithelial state) and X1 (mesenchymal state) displayed distinct upregulated genes. And the top five upregulated genes in X0 and X1 also exhibited high fold changes in X2 (intermediate state), demonstrating the intermediate state of X2. Moreover, hallmark gene set enrichment analysis of the differential genes between X0 and X1 revealed a significant enrichment of EMT‐related pathwaysin X1 (Figure 6f,g). All these results demonstrated a strong correspondence between three deconvolved malignant cell types and different EMT states.

Based on the dominant cell types predicted by SURF and the spatial information, we further investigated the EMT‐related cell‐cell interactions within the tissue using CellChat.^[^ ^39^ ^]^ The analysis of spatially resolved interaction strength revealed strong interactions between cell types in the cancerous region (X0: epithelial state, X1: mesenchymal state, X2: intermediate state) and those in the ECM region (X3: hepatic stellate cells, X4: fibroblast cells), highlighting the critical role of interactions between ECM and cancer cells in shaping and mediating tumor microenvironments (Figure 6h,i). Subsequently, we analyzed the interactions between the cancerous region and the ECM region within two key EMT‐related signaling pathway networks (CLDN pathway and CypA pathway), which are crucial for the initiation and regulation of EMT (Figure 6j).^[^ ^45^, ^46^, ^47^, ^48^ ^]^ In both pathways, we observed strong interactions between all three malignant cell types (X0, X1, and X2) and X3 (hepatic stellate cells). Moreover, malignant cell types X0 and X2 exhibited strong interactions with X4 (fibroblast cells). Ligand‐receptor pairs (CLDN3‐CLDN3, CLDN11‐CLDN11, and PPIA‐BSG) associated with ECM‐related cell types (X3, X4) and cancer‐related cell types (X0, X1, and X2) within these pathways were identified and visualized (Figure 6k,l). Notably, all three key ligands or receptors were distributed across both the cancer and ECM regions, indicating EMT‐related signaling activity in these areas. All these results highlighted SURF's capability to precisely resolve tumor heterogeneities and identify biologically relevant subtypes.

## 3 Discussion

The rapid advancement of ST technologies has revolutionized the study of the spatial architecture of biological tissues. However, the limited spatial resolution of spot‐level ST technologies necessitates the development of robust deconvolution algorithms to resolve cellular heterogeneity. Reference‐based deconvolution methods rely on matched single‐cell datasets, which are often challenging to acquire in real‐world scenarios due to sample availability, batch effects, and tissue heterogeneity. Reference‐free deconvolution algorithms address these limitations but remain relatively underexplored.

Here, we presented SURF, a self‐supervised deep learning algorithm specifically designed for reference‐free deconvolution. By leveraging a self‐supervised framework and introducing a distribution regulation loss, SURF adaptively extracts cell type‐specific information directly from ST data, eliminating the reliance on external scRNA‐seq references. SURF also employs a deep learning‐based autoencoder to capture the non‐linear gene interactions and applies contrastive learning to integrate the spot relationships, thereby enabling more comprehensive analysis and effective utilization of the hidden biological context information. Collectively, these strategies allow SURF to address limitations of existing methods and substantially improve deconvolution performance. Ablation studies further validated the contribution and necessity of each component to the overall performance (Figure S14, Supporting Information). In addition to estimating cell type proportions, SURF provides cell type‐specific gene expressions, thereby improving the interpretability of the deep learning model. These outputs can further be used for various biological analyses, including the identification of upregulated genes in each deconvolved cell type, cell‐cell communication analysis, and gene set enrichment analysis. Benchmarking of computational resources (Figure S15, Supporting Information) showed that SURF exhibited reasonable and comparable computation time and peak memory usage, indicating that its resource demands are well‐balanced and appropriate for practical applications. Notably, deep learning models are characterized by their exceptional flexibility and scalability. SURF, the sole deep learning‐based self‐supervised reference‐free model, stands out for its adaptability and suitability for further extensions and customizations, making it highly effective in addressing diverse research requirements.

SURF demonstrated robust deconvolution performance through rigorous benchmarking on both simulated and real datasets. It effectively deconvolved cell types in simulated ST data, outperforming reference‐free methods (CFS, STdeconvove, and BayesTME) and achieving competitive or superior performance compared to reference‐based methods. Notably, in scenarios lacking appropriate references—a common challenge, particularly in human samples—SURF consistently surpassed the performance of reference‐based methods. In real biological sample datasets, SURF also surpassed other reference‐free algorithms in a number of situations, encompassing different resolutions (10, 55, and 100 µm), species (mouse and human), spatial patterns (layer, block, and background), and disease status (normal and diseased). SURF precisely resolved the fine‐scale rostral migratory stream structure in the mouse main olfactory bulb dataset and captured seminiferous tubule structures in the mouse spermatogenesis dataset. In the human dorsolateral prefrontal cortex dataset, SURF distinctly characterized the multi‐layer structure. In the colorectal liver metastasis dataset, SURF successfully identified three epithelial‐to‐mesenchymal transition states within tumor regions.

Despite the advancement of sub‐cellular resolution ST technologies, their limitations—including a limited number of detected genes of certain methods, high costs, restricted accessibility, substantial computational demands, and challenges in managing large sample sizes—highlight the continued importance of multi‐cellular resolution ST platforms in both biological and clinical contexts. SURF represents a valuable and effective method, offering a reference‐free approach for delineating cellular compartments in multi‐cellular resolution ST data. However, SURF still has some limitations. SURF currently does not incorporate H&E staining images into the model, which have been demonstrated to be advantageous for certain ST‐related tasks.^[^ ^49^, ^50^, ^51^ ^]^ The integration of such data may further improve deconvolution performance. Moreover, SURF is presently limited to reference‐free deconvolution, without the option for reference‐based deconvolution when matched references are available.

SURF is a powerful method for reference‐free deconvolution that delineates cellular compartments in a self‐supervised manner by leveraging deep learning. It facilitates the investigation of tissue microenvironments and paves the way for understanding tissue structures in physiological and pathological contexts.

## 4 Experimental Section

### SURF Network Architecture—The Backbone of SURF

The backbone of SURF is a self‐supervised architecture (Figure 1), which takes the input of gene expression of each spot and outputs predictions of CTPs and deconvolved gene expressions of each cell type. For given ST data with *S* spots and *G* genes, let ***D*** be the *S* × *G* count matrix and ***L*** be the *S* × 2 coordinate matrix. First, the *G*‐dimensional gene expression vector of each spot ***d***_***s***_ is fed into the encoder to obtain a *K*‐dimensional CTPs prediction θ through the MLP layers, where *K* is the cell type number of the ST data.

(1) $$\theta =\mathrm{Softmax}\left(\mathrm{MLP}\left(d_{s}\right)\right)$$

The MLP layers of the encoder help to capture the complicated nonlinear interactions among genes. The details of MLP layers:

(2) $$\begin{array}{c} d_{t}=\mathrm{LeakyReLU}\left(\mathrm{BN}\left(\mathrm{Linear}\left(d_{s}\right)\right)\right)\\ d_{t}=\mathrm{Dropout}\left(d_{t}\right)\\ d_{t}=\mathrm{Linear}\left(d_{t}\right) \end{array}$$

where BN is a Batchnorm1d layer. The embedding dimensions of two linear layers are set as 32 and *K*, respectively. The dropout rate is set as 0.5. Then, CTP prediction ***θ*** is further input to the decoder to reconstruct the gene expression vector through a linear layer with softmax:

(3) $$\hat{d_{s}}=\mathrm{Softmax}\left(\beta \theta +b_{0}\right)$$

where $\hat{d_{s}}$ is the reconstructed gene expression vector. $\beta =[\beta_{1},\;\beta_{2},\;\text{…},\;\beta_{k}]$ is a *G* × *K* weight matrix of the linear layer and can be interpreted as the gene transcriptional profiles of *K* cell types. ***b***_0_ is a *G*‐dimensional bias vector of the Linear layer.

### The Reconstruction Loss

To ensure the encoder‐decoder architecture's ability to extract effective information, SURF employs a reconstruction loss *L_recon_* to constrain the input and output of the network to be as close as possible. *L_recon_* is designed based on cosine similarity and Kullback‐Leibler (KL) divergence.

(4) $$L_{cos}=1-\frac{d_{s}\cdot \;\hat{d_{s}}}{\left|d_{s}\right|\left|\hat{d_{s}}\right|}$$

(5) $$L_{KL}=\sum d_{s}\cdot \log\frac{d_{s}}{\hat{d_{s}}}$$

(6) $$L_{recon}=0.5\times \left(L_{cos}+L_{KL}\right)$$

### The Distribution Regulation Loss

Previous studies have shown that the Dirichlet distribution can be used to model CTPs.^[^ ^18^, ^20^ ^]^ To introduce this prior knowledge into the model, SURF designs a distribution regulation loss with the Maximum Mean Discrepancy (MMD) distance,^[^ ^52^, ^53^ ^]^ which is inspired by neural topic models.^[^ ^54^, ^55^ ^]^ The definition of distribution regulation loss is as follows:

(7) $$L_{distri-reg}=\mathrm{MMD}\left(Q_{\Theta },\;P_{\Theta }\right)$$

(8) $$\begin{array}{ccc} \mathrm{MMD}\left(Q_{\Theta },\;P_{\Theta }\right) & = & \frac{1}{N\left(N-1\right)}\sum_{i\neq j}k\left(\theta_{i},\;\theta_{j}\right)+\frac{1}{N\left(N-1\right)}\sum_{i\neq j}k\left(\theta_{i}^{\prime },\;\theta_{j}^{\prime }\right)\\ & & -\frac{2}{N^{2}}\sum_{i,\;j}k\left(\theta_{i},\;\theta_{j}^{\prime }\right) \end{array}$$

(9) $$k\left(a,\;b\right)=\exp\left(-\frac{\mathrm{arcco}s^{2}\left(\sqrt{a\cdot b}\right)}{t}\right)$$

where ***θ***_***i***_ (i=1,2,…,N) represents the CTPs prediction of a batch, which forms the predicted distribution ***Q***_**Θ**_, and ***θ***_***i***_′ (i=1,2,…,N) is sampled from Dirichlet distribution ***P***_**Θ**_. *N* is the batch size, and *t* is a scale parameter, which is set as 0.1 in SURF. The distribution regulation loss calculates the MMD distance between the prediction distribution and the Dirichlet distribution. The CTPs predictions of SURF get closer to the Dirichlet distribution with the decline of the distribution regulation loss during model iterations.

### The Biologically Informed Contrastive Loss

Unlike existing reference‐free deconvolution methods that ignore spot relationships or only utilize spatial distance to define spot relationships, SURF leverages both spatial distance and gene expressions to define spot relationships and integrates them into the model using contrastive learning. Spatially adjacent spots are likely to possess similar cell type compositions,^[^ ^24^, ^25^, ^26^ ^]^ indicating close relationships. In contrast, spots with significant gene expression disparities tend to have distinct cellular constituents, representing distant relationships. To conduct contrastive learning, SURF selects adjacent spots with high gene expression similarity to the focal spot as positive samples, and selects those with the lowest expression similarity across the tissue as negative samples (more details in the next section). After selecting the positive and negative samples, the focal spot and its corresponding positive and negative samples are input into the encoder to obtain the CTPs predictions ***θ***, ***θ*** ^+^, and ***θ*** ^−^ respectively. Then, SURF modifies these CTP predictions with a biologically informed contrastive loss:

(10) $$L_{contras}=\max\left(\gamma ,0\right)$$

(11) $$\begin{array}{c} \gamma =\frac{1}{N^{-}}\sum_{i=1}^{N^{-}}\mathrm{weighted}\_\mathrm{cosine}\left(\theta ,\;\theta_{i}^{-}\right)\\ -\frac{1}{N^{+}}\sum_{i=1}^{N^{+}}\mathrm{weighted}\_\mathrm{cosine}\left(\theta ,\;\theta_{i}^{+}\right)+\varepsilon \end{array}$$

(12) $$\mathrm{weighted}\_\mathrm{cosine}\left(\theta_{1},\;\theta_{2}\right)=\frac{\sum_{i=1}^{K}w_{i}\theta_{1i}\theta_{2i}}{\left|\theta_{1}\right|\left|\theta_{2}\right|}$$

where *w_i_* (i=1,2,…,K) is the weight assigned to each cell type and can be adaptively adjusted during the model fitting process without manual intervention. ε is the margin value with a default value of 0.05. As the biologically informed contrastive loss function decreases during the model fitting process, ***θ*** and ***θ*** ^+^ become closer while ***θ*** and ***θ*** ^−^ become further apart.

### The Selection of Positive and Negative Samples

The selection strategy of positive and negative samples is as follows. First, neighboring spots are defined as those closest to the focal spot in spatial coordinates. For regular spatial modes in square shape (such as ST technology) (Figure S16a, Supporting Information) and regular spatial modes in hexagonal shape (such as Visium technology) (Figure S16b, Supporting Information), neighboring spots are a circle of spots around the focal spot. For irregular spatial modes like slide‐seq technology (Figure S16c, Supporting Information), neighboring spots are the top ten nearest spots of the focal spot.

Then, the positive samples are selected, which exhibited close relationships with the focal spot. Let *B* be the total number of neighboring spots of all spots. The cosine similarities between the gene expressions of focal spots and their neighboring spots are calculated. Then all these cosine similarity values are put together and sort them in reverse order, and set the value with the ranking of *R_p_* = B*η as the positive threshold. η was set as 0.05 empirically in all experiments. For a given focal spot, neighboring spots with cosine similarity beyond the positive threshold were defined as positive samples.

Next, the negative samples are selected, which exhibited distant relationships with the focal spot. Let *S* be the total number of spots in the ST dataset. The cosine similarities between the gene expressions of focal spots and all other spots of the tissue are calculated. Then, all these S*S cosine similarity values are put together and sorted in ascending order, and set the value with the ranking of *Ranking* = S*S*σ as the negative threshold. σ was set as 0.05 empirically in all experiments. For a given focal spot, spots with cosine similarity below the negative threshold were defined as negative samples. To avoid too many negative samples increasing the computational burden, an upper limit on the number of negative samples neg_num for each spot is set. The neg_num is set as neg_num=max{S∗σ,200}

### The Total Loss Function

The biologically informed contrastive module worked together with the backbone, and the total loss function is:

(13) $$L=L_{recon}+\lambda L_{distri-reg}+L_{contras}$$

where λ is the weight for *L*_*distri* − *reg*_ and is set as 10. In all the experiments, SURF iterates 500 epochs with the AdamW optimizer, setting the learning rate to 0.01 for the initial 200 epochs and adjusting it to 0.001 for the remaining epochs. The batch size is set as 64 by default. The SURF model is based on Pytorch implementation. The hyperparameters were empirically optimized using grid search to determine values that yielded good performance across the simulated datasets in our study.

### Cell Type Annotation

To match the deconvolved cell types with the true cell types, the Pearson correlation coefficients were calculated between the deconvolved and the true cell type transcriptional profiles when paired single‐cell references were available. Each deconvolved cell type was assigned to the true cell type with the highest Pearson correlation value. In real datasets, where matched single‐cell references were challenging to obtain, marker genes identified in relevant studies and tools such as CellMarker 2.0,^[^ ^56^ ^]^ DISCO,^[^ ^57^ ^]^ and PanglaoDB^[^ ^58^ ^]^ were ultilized to annotate the deconvolved cell types.

### Cell Type Number Selection

The cell type number should be chosen for the model. Inspired by STdeconvolve, we used two indices, representation discrepancy (RD) and rare cell type number, to determine the optimal cell type number *K*. In the context of SURF, RD measures the difference between the model's reconstructed gene expression and the original gene expression, which is defined as the exponential form of cross‐entropy loss:

(14) $$\mathrm{RD}=\exp\left\{-\frac{\sum_{s=1}^{S}\sum_{i=1}^{G}d_{si}\log\left(\hat{d_{si}}\right)}{S}\right\}$$

where *d_si_* and $\hat{d_{si}}$ represent the original and reconstructed expression value of the i‐th gene in the s‐th spot, respectively. *S* represents the total number of spots, and *G* represents the total number of genes. A lower RD means a better representation of the dataset. Rare cell type number refers to the number of deconvolved cell types with a mean cell type proportion less than 5% across all spots. Such rare cell types are often considered to be irrelevant or spurious subdivisions of primary cell types. A high rare cell type number indicates the unreliability of the model. As cell type number *K* increases, RD often exhibits a declining trend, and the rare cell type number shows an upward trend. There is a trade‐off between RD and the rare cell type number. The optimal cell type number *K* is determined at the point where it reaches the lowest RD while minimizing the rare cell type number. Moreover, the user can choose the optimal *K* with the help of their biological knowledge.

### Synthetic ST Data Generation

A human pancreas single‐cell dataset with cell type annotations was used to generate simulated ST data.^[^ ^27^ ^]^ To guarantee the quality of the simulation, cell types whose number of cells accounted for less than 1% of the total cell number in the scRNA‐seq dataset were excluded. Eight cell types remained and were employed in the simulation. Two sets of simulated data with three different spatial patterns (layer pattern, block pattern, and background pattern) were simulated to evaluate deconvolution methods in different scenarios. For the first set, within each spatial pattern, six cell types were randomly selected, with each cell type predominant in a specific subregion. For the second set, more complex scenarios were modeled. Within each spatial pattern, five cell types were randomly selected, with each dominating a different subregion. Additionally, three more cell types were introduced as dispersed cell types, each distributed across all tissue regions. The simulation of each spot is detailed in Supporting Methods.

### Benchmark Metrics

To compare the performance of SURF with other deconvolution algorithms in simulated datasets, PCC and RMSE between the predicted CTPs and ground truth CTPs for each spot were calculated. To compare the performance of SURF with other reference‐free deconvolution algorithms in real datasets, the mean expression of marker genes and ARI were calculated. The detailed definitions of benchmark metrics are provided in the Supporting Methods.

### Method Comparison

SURF was compared with five reference‐based and three reference‐free deconvolution methods. Reference‐based methods included SONAR, Cell2location, Redeconve, DestVI, DSTG, and GraphST. Reference‐free methods included BayeTME, STdeconvolve, and CFS. For all methods, the tutorials were followed and the recommended parameters were used for deconvolution. All experiments were repeated five times, and the best results are shown. For SONAR, the bandwidth was set as 1.2 times the minimal distance. For Cell2location, the “N_cells_per_location” was set according to different datasets, and the “detection_alpha” was set to 20. When performing Redeconve, the default mode was used to choose hyperparameters. In addition, the down‐sampling strategy provided by Redeconve was adopted to alleviate the heavy computational burden caused by large single‐cell datasets. For DestVI, the maximum training epochs were set as 2000 for both reference data and ST data. For DSTG, we set the learning rate = 0.01 and epochs = 1000. For GraphST, we set the epochs = 1200. For BayesTME, a suitable smoothness hyperparameter λ for each dataset was manually selected and then used the “phenotype_selection” function to select the best number of cell types. STdeconvolve provided three approaches to choosing the number of cell types. All three approaches were implemented and the optimal number of cell types with the best metrics was picked for each dataset. When performing CFS, we set the variable_features = 2000 and used the default “icafast” method. All experiments were conducted using two devices. The first device is a Linux server equipped with an AMD EPYC 7763 CPU and an NVIDIA A800 GPU. The second device is a Windows workstation with an Intel Xeon Silver 4215R CPU and an NVIDIA RTX 3090 GPU.

### Computational Resources Comparison

The computation time and peak memory usage of different deconvolution methods were measured in three real datasets, with spot numbers ranging from several hundred to tens of thousands. BayesTME was not applied to the mouse spermatogenesis dataset due to its heavy computational burden. Although DSTG is a deep learning‐based model, its TensorFlow implementation was not compatible with our system, so it was ran on the CPU instead. All experiments were conducted on a Linux server utilizing an AMD EPYC 7763 CPU and an NVIDIA A800 GPU.

### Statistical Analysis

For the count matrix of ST data, spot outliers with insufficient gene counts were first excluded. By default, spots with no more than 100 gene counts were filtered out. Subsequently, gene filtering was performed, removing genes present in all the spots or detected in fewer than 5% of spots. Next, SURF identified significantly overdispersed genes.^[^ ^59^ ^]^ After the above steps, if there are still too many genes, the top 1000 most overdispersed genes were selected by default. Finally, the total gene counts of each spot were scaled to 1 and then fed into the network. Python and R were used for statistical analysis.

## Conflict of Interest

The authors declare no conflict of interest.

## Code Availability

An open‐source Python implementation of SURF is accessible at https://github.com/lllsssyyyy/SURF.

## Supporting information



Supporting Information

## Data Availability

The human pancreas dataset used in the simulated ST data of different spatial patterns is available at https://www.ncbi.nlm.nih.gov/geo/query/acc.cgi?acc=GSE84133.^[^ ^27^ ^]^ The data of ‘human3’ was used to generate the simulated ST data, and the data of ‘human4’ was used as a real single‐cell reference. The mouse main olfactory bulb ST dataset is available at https://www.spatialresearch.org/resources‐published‐datasets/doi‐10‐1126science‐aaf2403.^[^ ^11^ ^]^ The mouse olfactory bulb single‐cell dataset is available at https://www.ncbi.nlm.nih.gov/geo/query/acc.cgi?acc=GSE121891.^[^ ^28^ ^]^ The mouse spermatogenesis ST dataset is available at https://www.dropbox.com/s/ygzpj0d0oh67br0/Testis_Slideseq_Data.zip?dl=0&e&1&file_subpath&%2FData%2FWT+Slide‐seq+data%2FWT1.^[^ ^33^ ^]^ The mouse germ cell single‐cell dataset is available at https://www.ebi.ac.uk/biostudies/ArrayExpress/studies/E‐MTAB‐6946?query=spermatogenesis.^[^ ^35^ ^]^ The human dorsolateral prefrontal cortex dataset is available at https://zenodo.org/records/6925603#.YuM5WXZBwuU.^[^ ^36^ ^]^ The human post‐mortem brain single‐cell dataset is available at https://www.ncbi.nlm.nih.gov/geo/query/acc.cgi?acc=GSE144136.^[^ ^40^ ^]^ The human colorectal liver metastasis dataset is available at https://www.ncbi.nlm.nih.gov/geo/query/acc.cgi?acc=GSE206552.^[^ ^41^ ^]^
