# Supplementary material for: SURF: A Self‐Supervised Deep Learning Method for Reference‐Free Deconvolution in Spatial Transcriptomics
Source: Adv Sci (Weinh). 2025 Aug 26;12(43):e05456. doi: 10.1002/advs.202505456 (PMC12631812; doi:10.1002/advs.202505456)
Supplement: Supplementary file 1 — Supporting Information [file ADVS-12-e05456-s001.pdf]

Supporting Information

SURF: a self-supervised deep learning method for reference-free deconvolution in spatial transcriptomics

*Shuyu Liang[1], Zixia Zhou[2], Peng Huang[1], Junhu Fu[1], Jing Jiao[1], Yunxia Huang[3, 4], Shichong Zhou[3, 4], Guanlin Wang[5, 6, *], Yuanyuan Wang[1, 7, *], Yi Guo[1, 7, *]*

1 School of Information Science and Technology, Fudan University, Shanghai 200433, China
2 Department of Radiation Oncology, Stanford University, Stanford, CA 94305, USA
3 Department of Ultrasound, Fudan University Shanghai Cancer Center, Shanghai 200032, China
4 Department of Oncology, Shanghai Medical College, Fudan University, Shanghai 200032, China
5 Shanghai Key Laboratory of Metabolic Remodeling and Health, Institute of Metabolism and Integrative Biology, Centre for Evolutionary Biology, Fudan University, Shanghai 200032, China
6 Shanghai Qi Zhi Institute, Shanghai 200032, China
7 Key Laboratory of Medical Imaging Computing and Computer Assisted Intervention of Shanghai, Shanghai 200032, China
* Correspondence: guanlin_wang@fudan.edu.cn (G.W.), yywang@fudan.edu.cn (Y.W.), guoyi@fudan.edu.cn (Y. G.).

Supplementary Figures

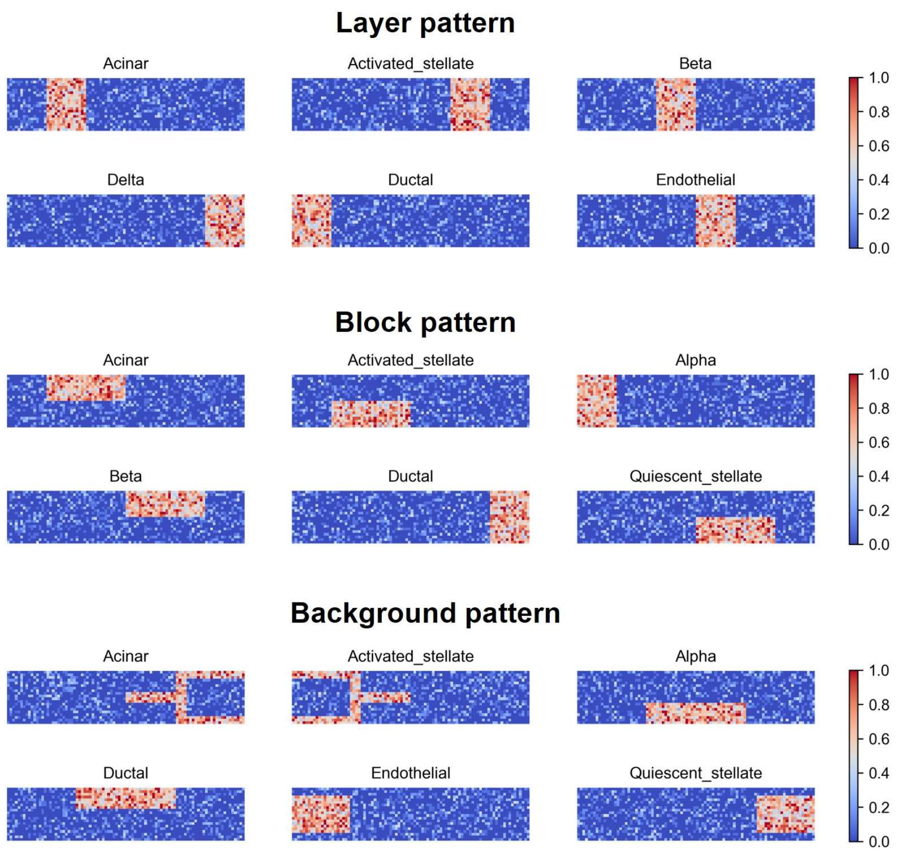

Figure S1. Ground truth cell type proportions in the first group of simulated datasets with three different spatial patterns. a Layer pattern. **b** Block pattern. **c** Background pattern. The color of each spot represents the proportion of each cell type.

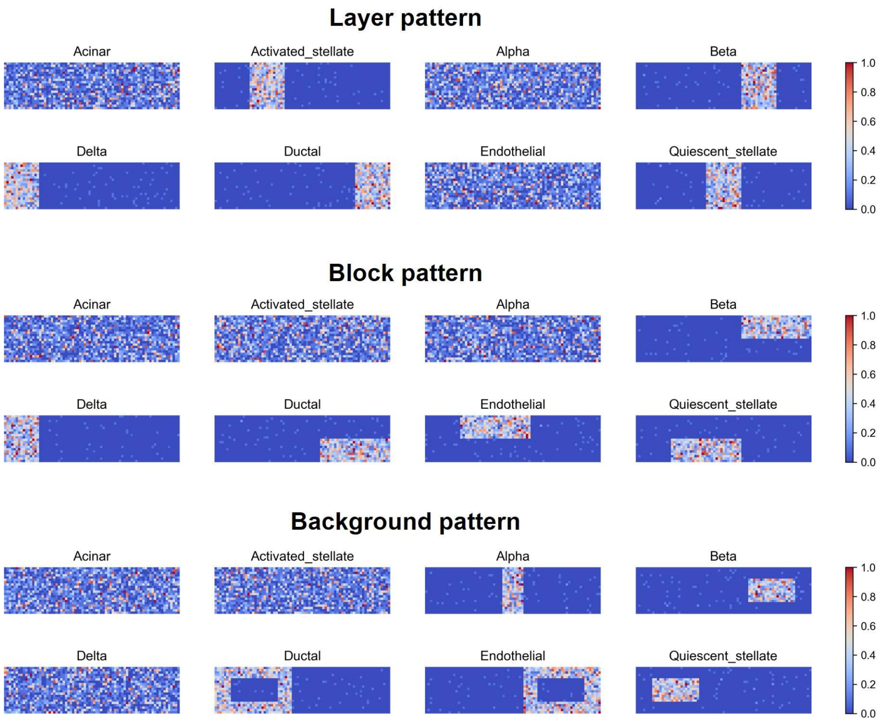

Figure S2. Ground truth cell type proportions in the second group of simulated spatial transcriptomics data with three different spatial patterns. a Layer pattern. **b** Block pattern. **c** Background pattern. The color of each spot represents the proportion of each cell type.

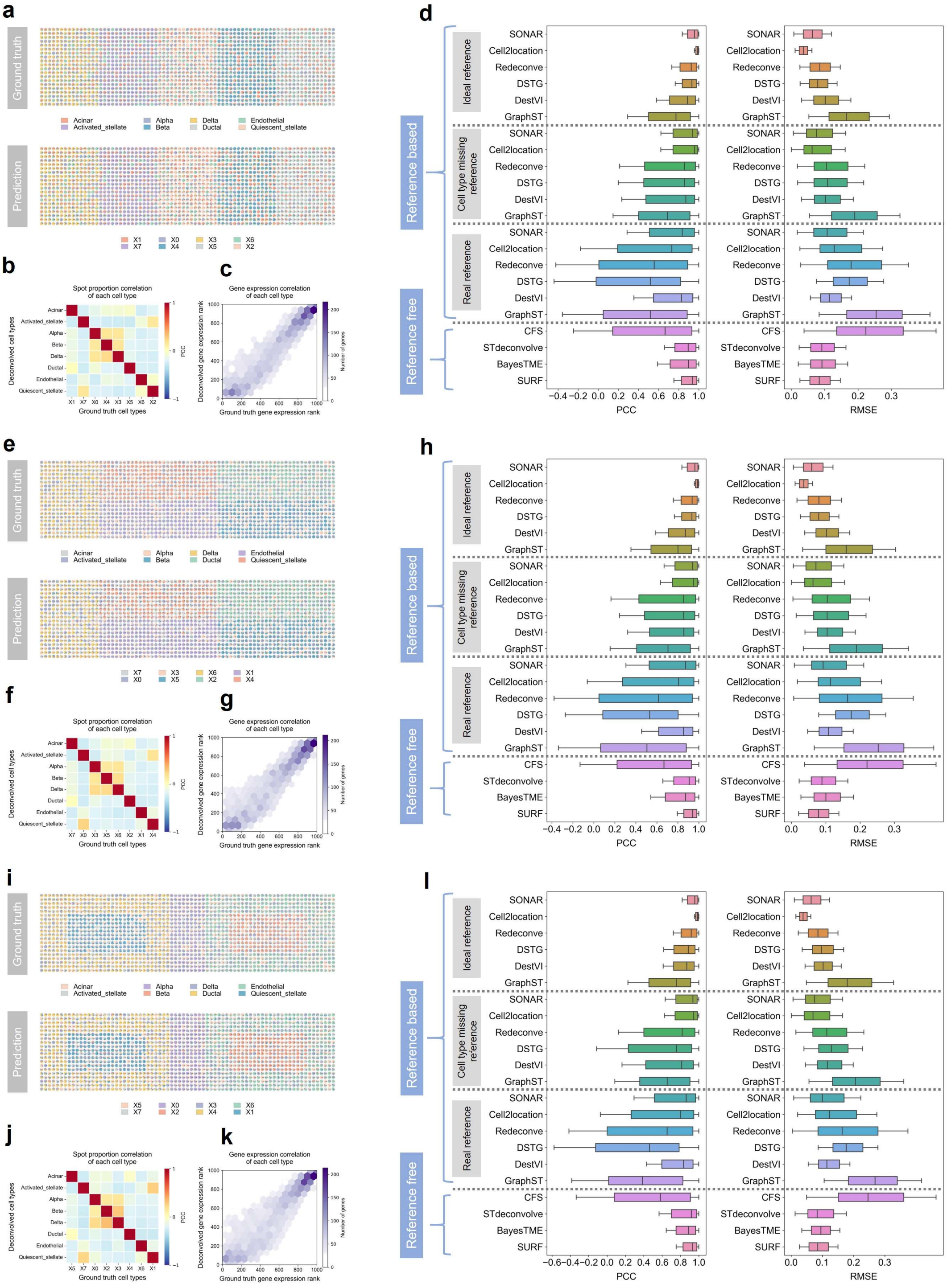

Figure S3. Benchmarking in the second set of simulated spatial transcriptomics data with layer pattern (a-d), block pattern (e-h), and background pattern (i-l). a, e, i Ground truth (top) and SURF predictions (bottom) of cell type proportions in different spatial patterns. The pie chart at each spot represents the cell type proportions. **b, f, j** Pearson correlations between the deconvolved and ground truth cell type proportions of different cell types. **c, g, k** Gene expression rankings in the deconvolved cell type transcriptional profiles compared to gene expression rankings in the ground truth cell type transcriptional profiles. The color of the hexagon represents the number

of genes located in this region (only hexagons with 5 or more genes are shown). **d, h, l** Boxplots of Pearson correlation coefficient (PCC) and root mean square error (RMSE) of different deconvolution methods. Center line, median value; box limits, upper and lower quartiles; whiskers, 0.5× interquartile range. All the methods are divided into two categories: reference-based and reference-free. Reference-based methods using ideal reference (top), cell type missing reference (middle), and real reference (bottom) are shown respectively.

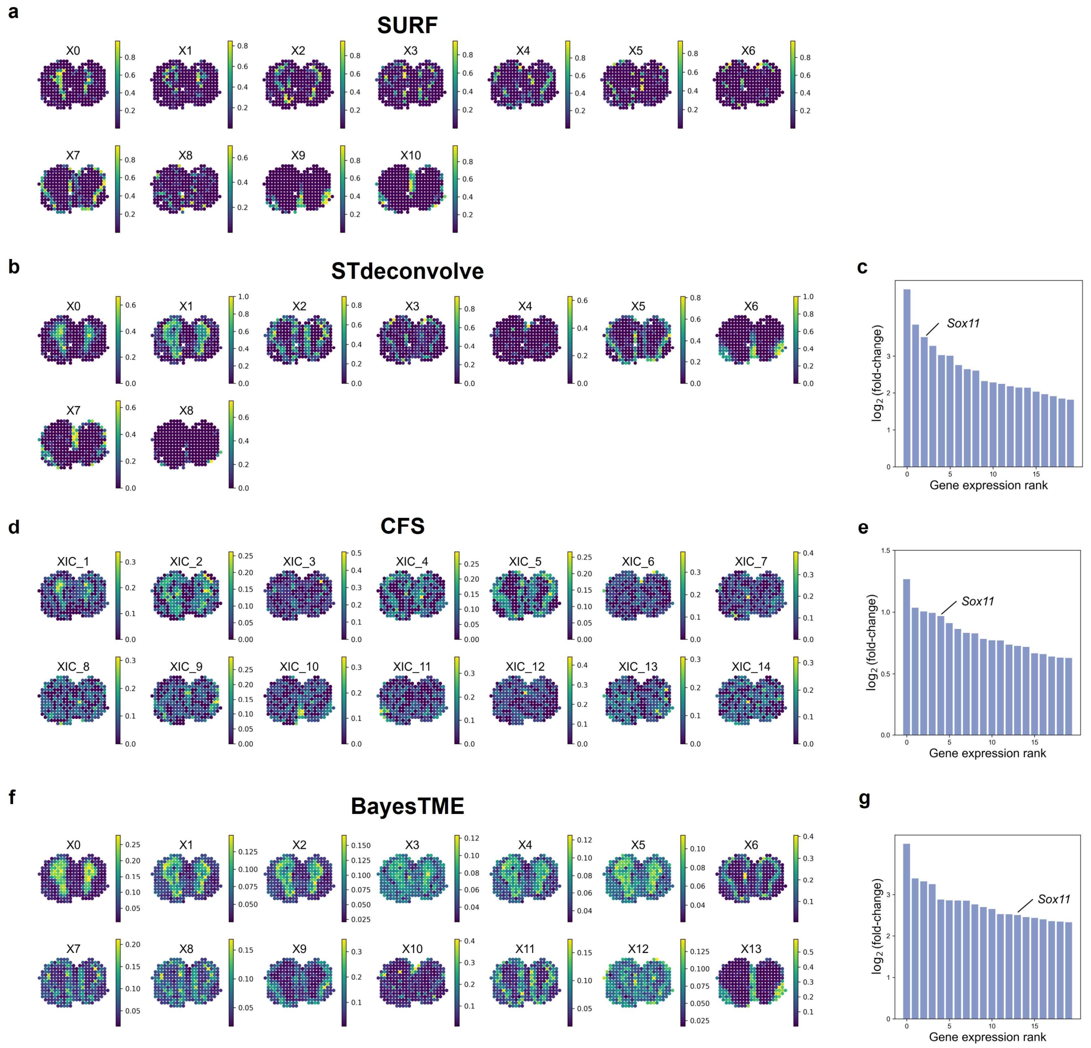

Figure S4. Deconvolution results of reference-free methods on the mouse main olfactory bulb dataset. a Deconvolved cell type proportions of SURF. The color of each spot represents the proportion of each cell type. **b** Deconvolved cell type proportions of STdeconvolve. The color of each spot represents the proportion of each cell type. **c** Top twenty upregulated genes of the rostral migratory stream (RMS)-related cell type predicted by STdeconvolve. **d** Deconvolved cell type proportions of CFS. The color of each spot represents the proportion of each cell type. **e** Top twenty upregulated genes of the RMS-related cell type predicted by CFS. **f** Deconvolved cell type proportions of BayesTME. The color of each spot represents the proportion of each cell type. **g** Top twenty upregulated genes of the RMS-related cell type predicted by BayesTME.

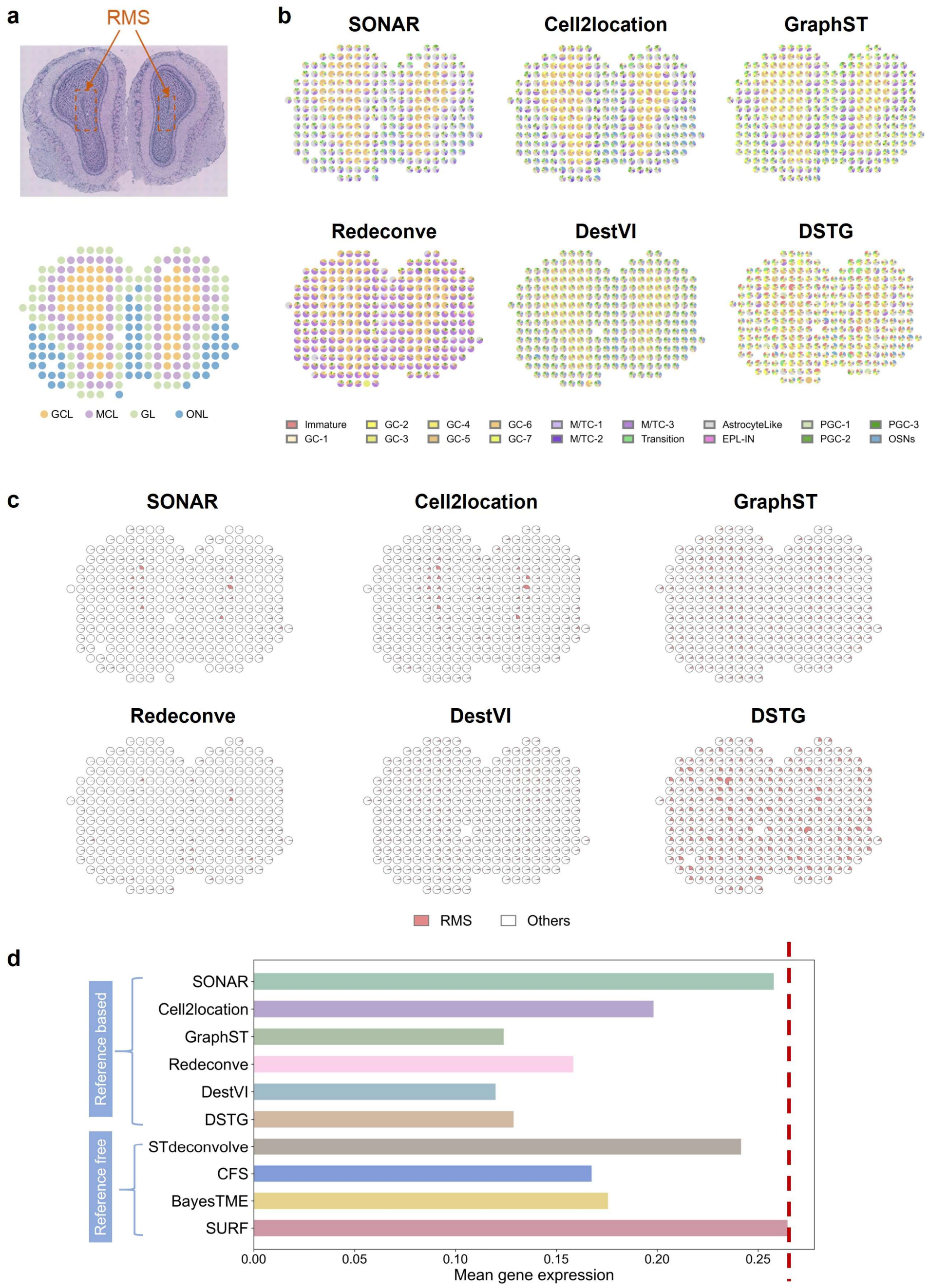

Figure S5. Comparison with reference-based methods on the mouse main olfactory bulb dataset. a H&E staining image and spot-level annotations of the mouse main olfactory bulb dataset.[1] The red box indicates the area where the rostral migratory stream (RMS) is located. **b** Deconvolution results of different reference-based methods. The pie chart at each spot represents the cell type proportions of this spot. (GC, granule cell; M/TC, mitral and tufted cell; EPL-IN, external plexiform layer interneuron; PGC, periglomerular cell; OSN, olfactory sensory neuron) **c** Distributions of the RMS-related cell type identified by different reference-based methods. **d** Mean expression of marker genes *Sox11* in the RMS-related cell type of different deconvolution methods.

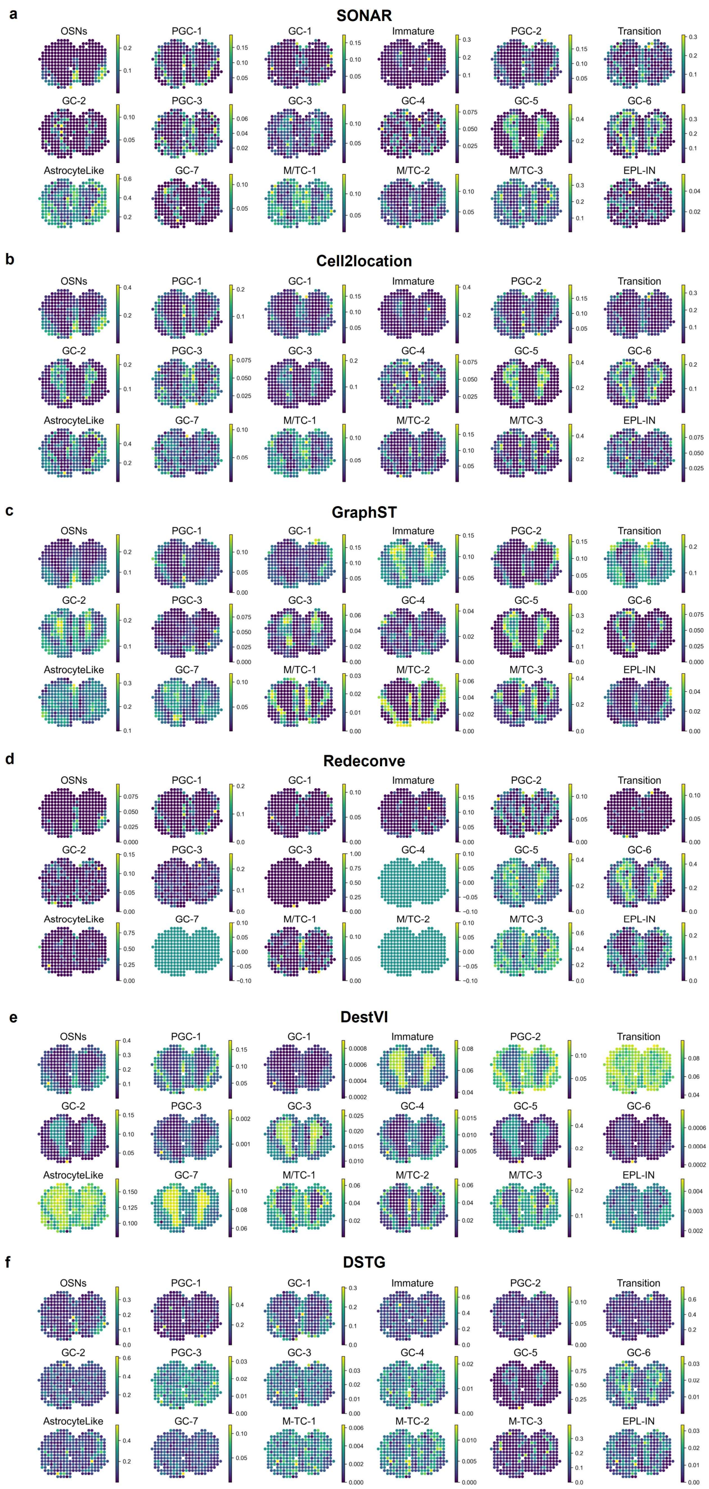

Figure S6. Deconvolution results of reference-based methods on the mouse main olfactory bulb dataset. a Deconvolved cell type proportions of SONAR. **b** Deconvolved cell type proportions of Cell2location. **c** Deconvolved cell type proportions of GraphST. **d** Deconvolved cell type proportions of Redeconve. **e** Deconvolved cell type proportions of DestVI. **f** Deconvolved cell type proportions of DSTG. The color of each spot represents the proportion of each cell type.

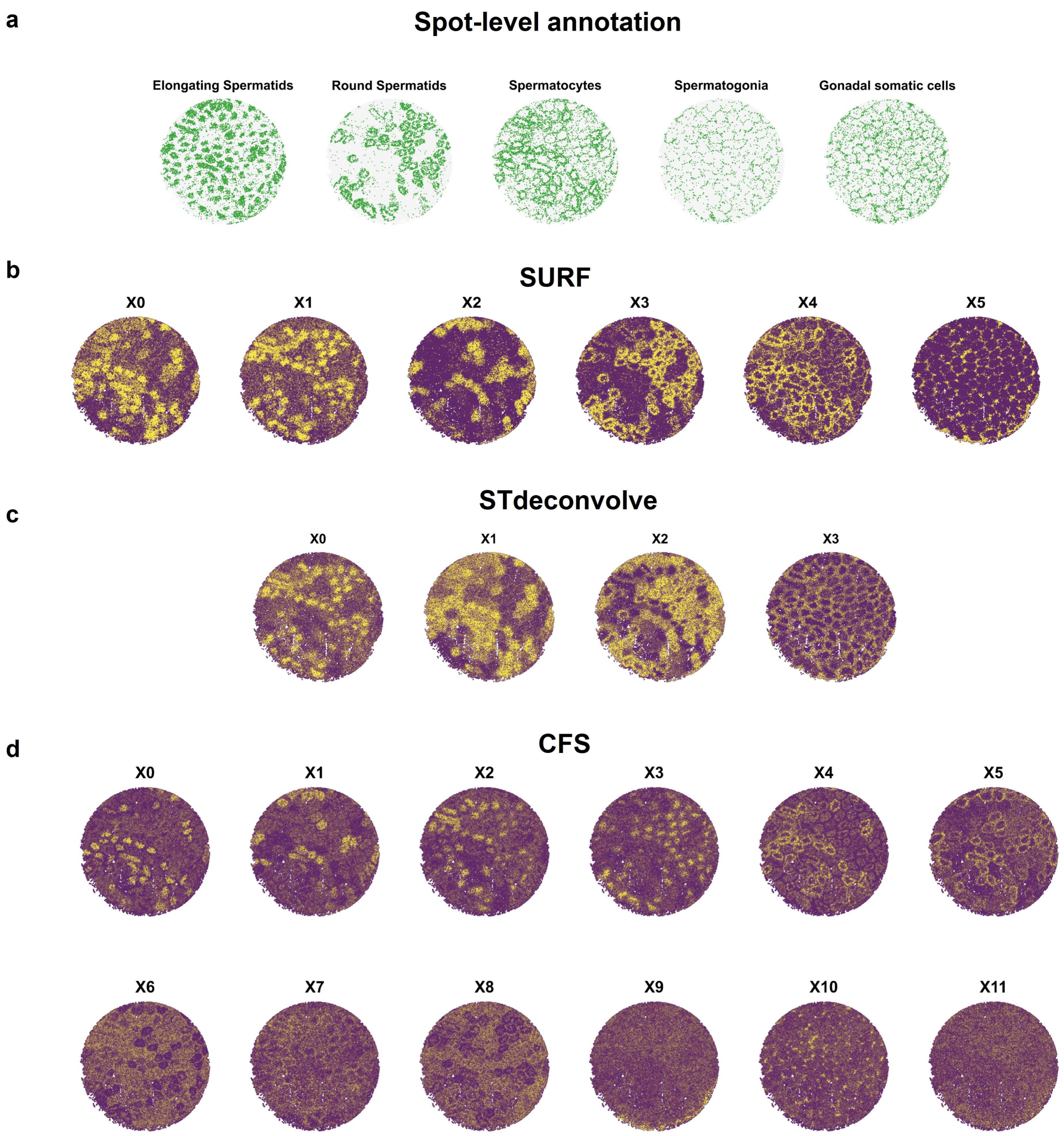

Figure S7. Deconvolution results of reference-free methods on the mouse spermatogenesis dataset. a Spot-level annotation of cell types. **b** Deconvolved cell type proportions of SURF. **c** Deconvolved cell type proportions of STdeconvolve. **d** Deconvolved cell type proportions of CFS. The color of each spot represents the proportion of each cell type.

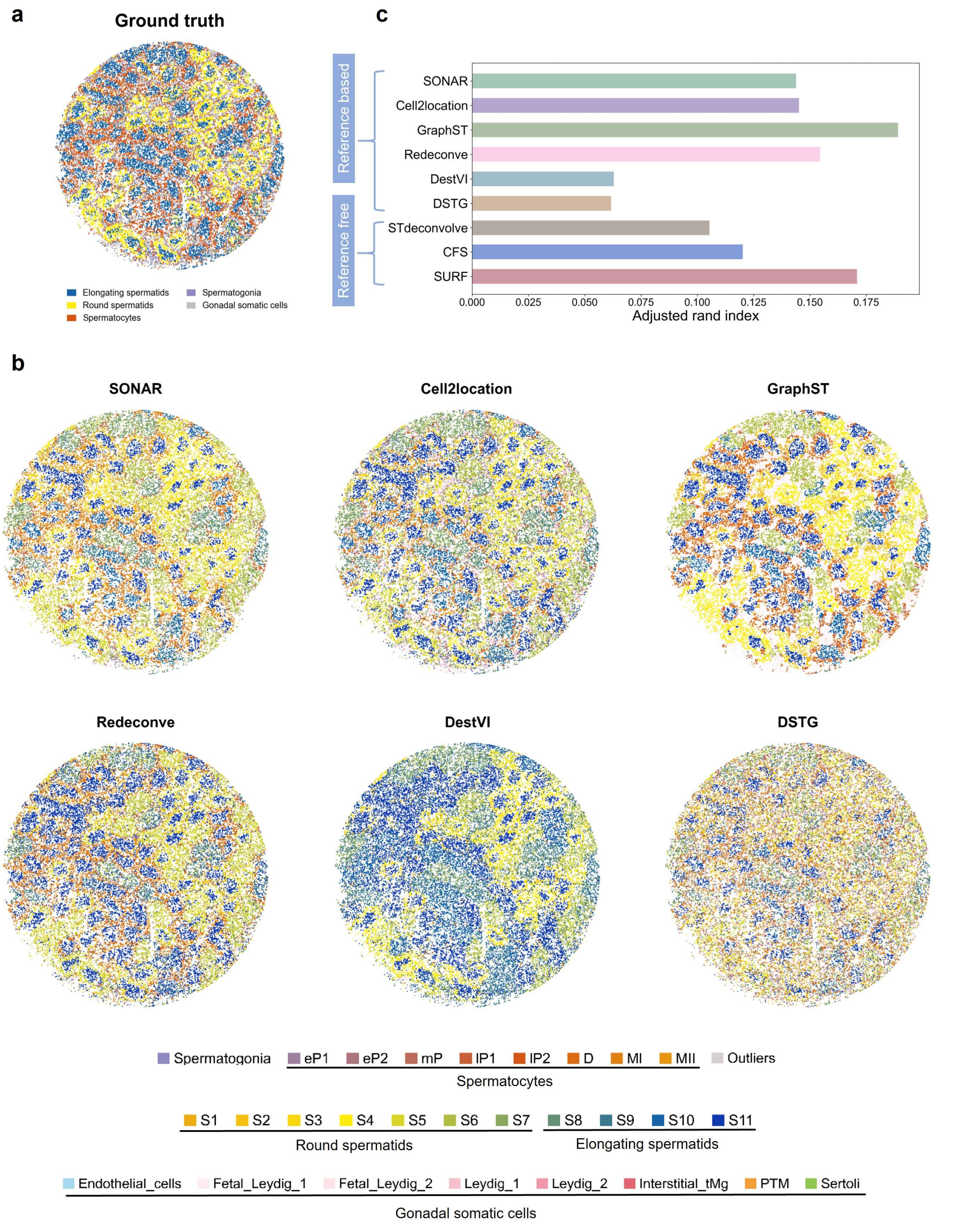

Figure S8. Comparison with reference-based methods on the mouse spermatogenesis dataset. **a** Spot-level annotations of the mouse spermatogenesis dataset. **b** Deconvolution results of different reference-based deconvolution methods. The color at each spot represents the dominant cell type of this spot. (eP: early-pachytene spermatocyte; mP: mid-pachytene spermatocyte; lP: late-pachytene spermatocyte; D: diplotene spermatocyte; MI: meiosis I; MII: meiosis II, S1-S11: step 1-11 spermatids; Interstitial_tMg: Interstitial testicular macrophages; PTM: peritubular myoid cells) **c** Adjusted rand index between dominant cell type predictions and spot-level annotations of different deconvolution methods.

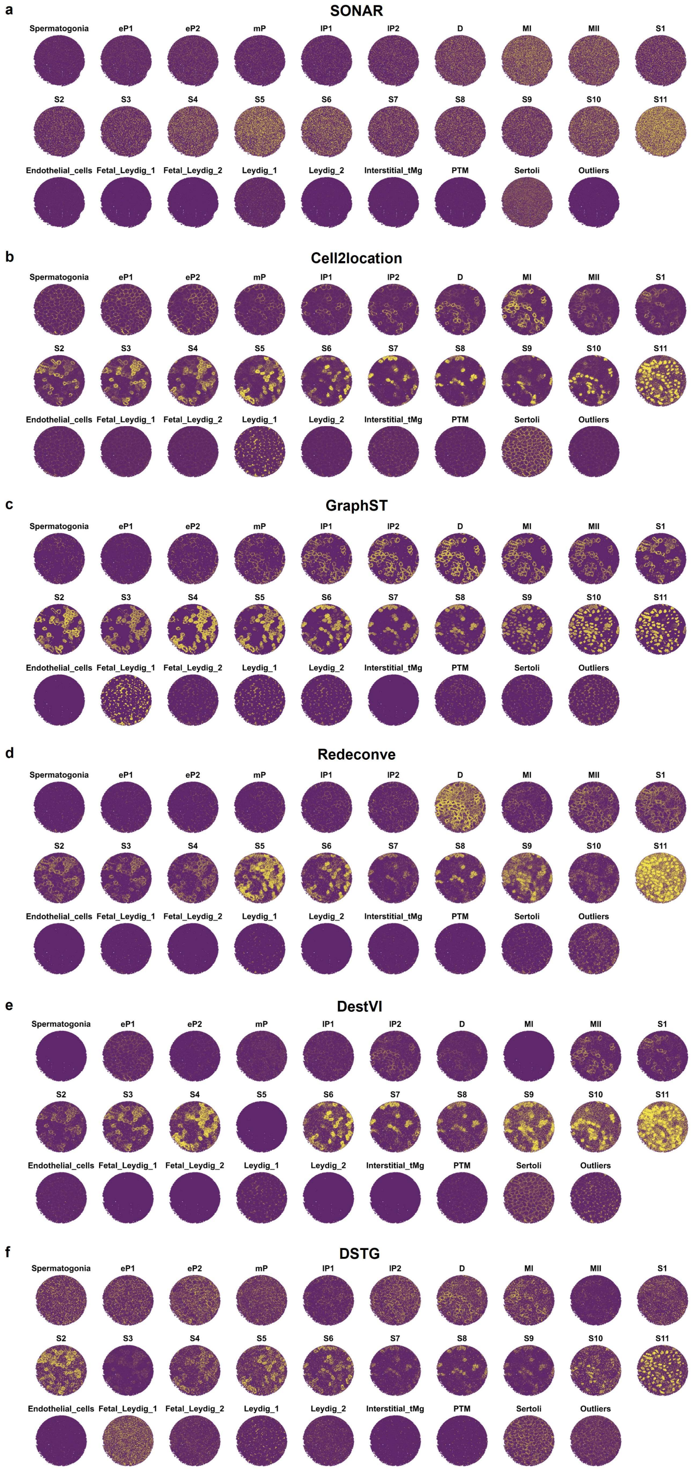

Figure S9. Deconvolution results of reference-based methods on the mouse spermatogenesis dataset. a Deconvolved cell type proportions of SONAR. **b** Deconvolved cell type proportions of Cell2location. **c** Deconvolved cell type proportions of GraphST. **d** Deconvolved cell type proportions of Redeconve. **e** Deconvolved cell type proportions of DestVI. **f** Deconvolved cell type proportions of DSTG. The color of each spot represents the proportion of each cell type.

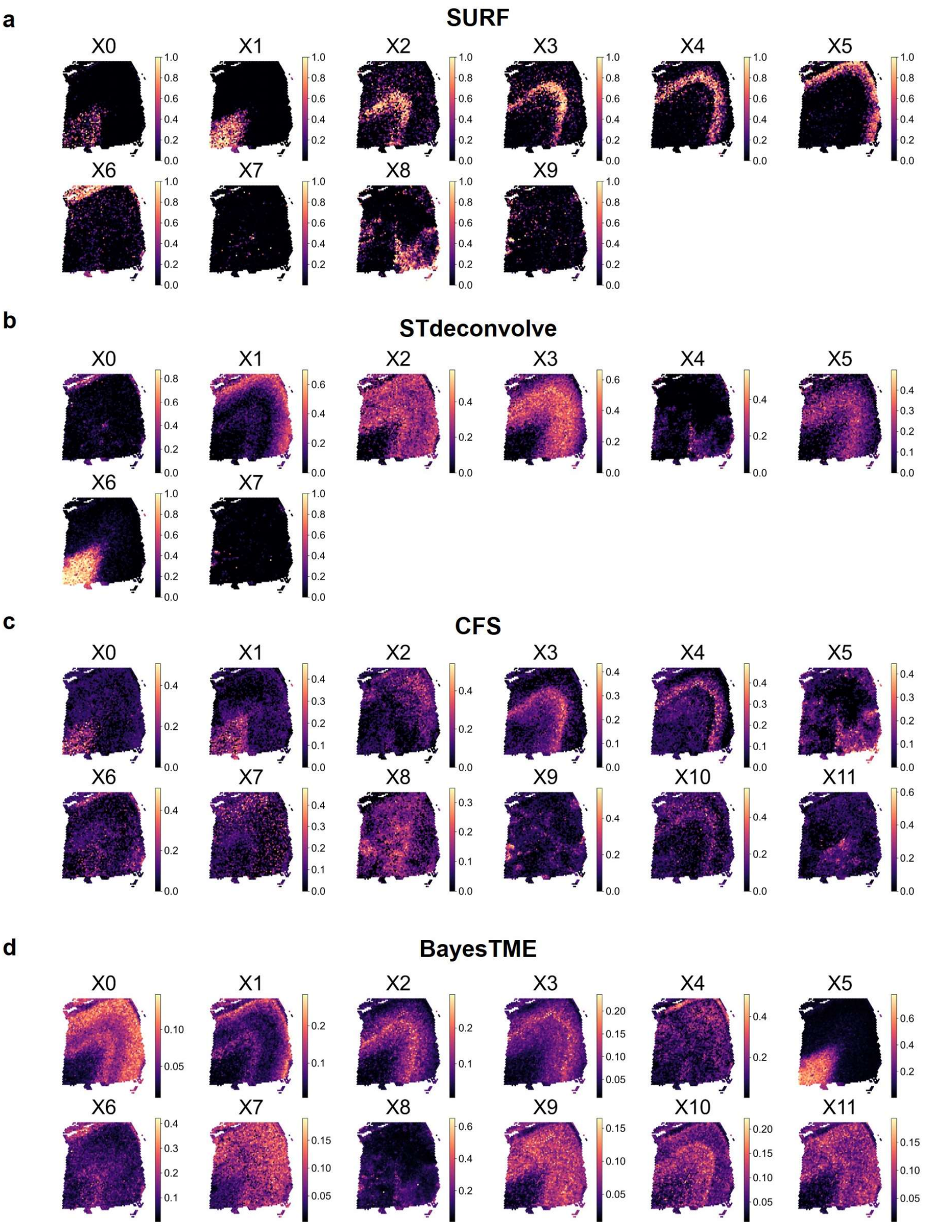

Figure S10. Deconvolution results of reference-free methods on the human dorsolateral prefrontal cortex dataset. a Deconvolved cell type proportions of SURF. **b** Deconvolved cell type proportions of STdeconvolve. **c** Deconvolved cell type proportions of CFS. **d** Deconvolved cell type proportions of BayesTME. The color of each spot represents the proportion of each cell type.

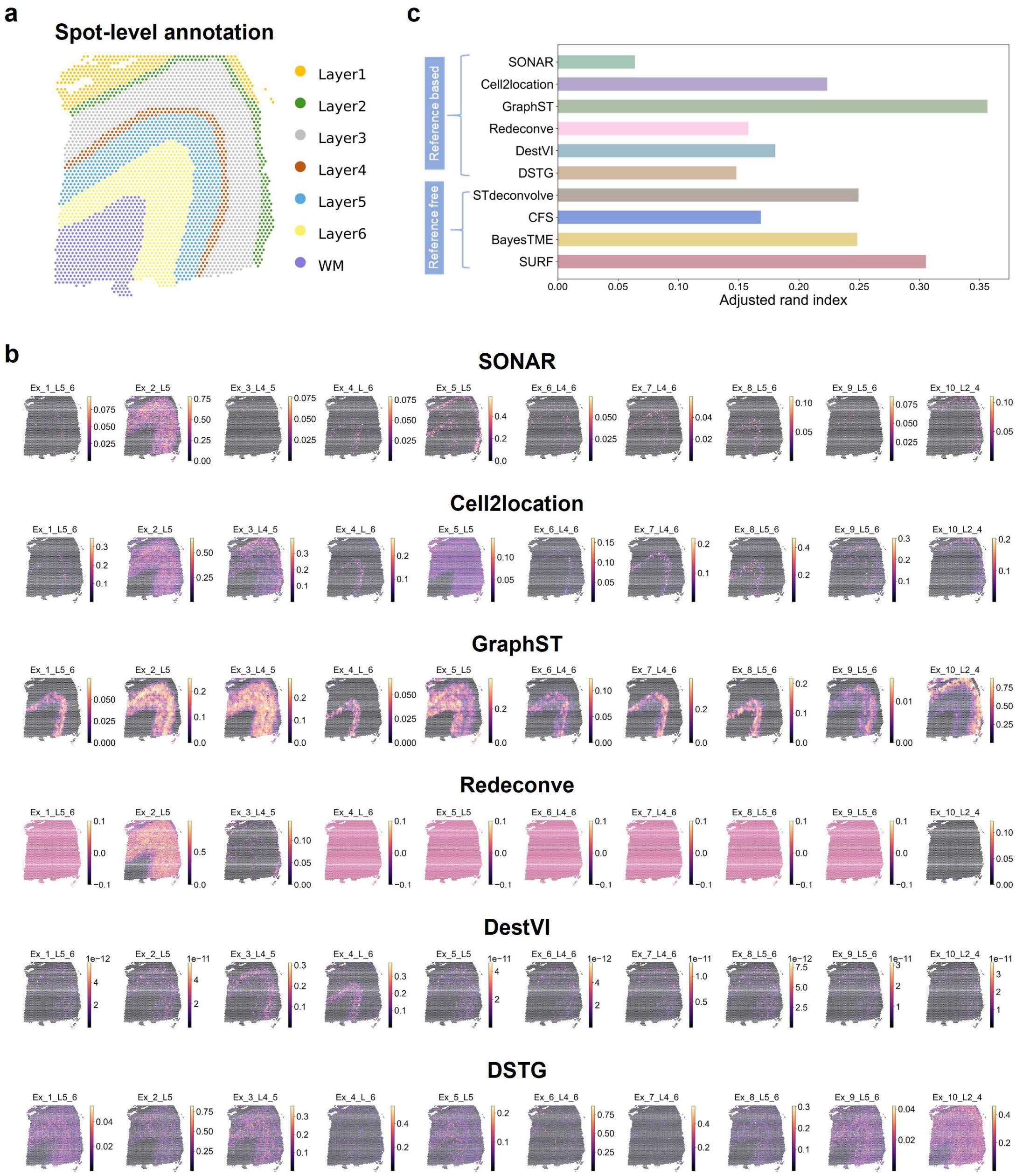

Figure S11. Comparison with reference-based methods on the human dorsolateral prefrontal cortex dataset. a Spot-level annotations of the human dorsolateral prefrontal cortex dataset. **b** Deconvolved cell type proportions of major neuronal cell types of different reference-based methods. (Ex: excitatory neuron; L: layer) **c** Adjusted rand index between dominant cell type predictions and spot-level annotations of different deconvolution methods.

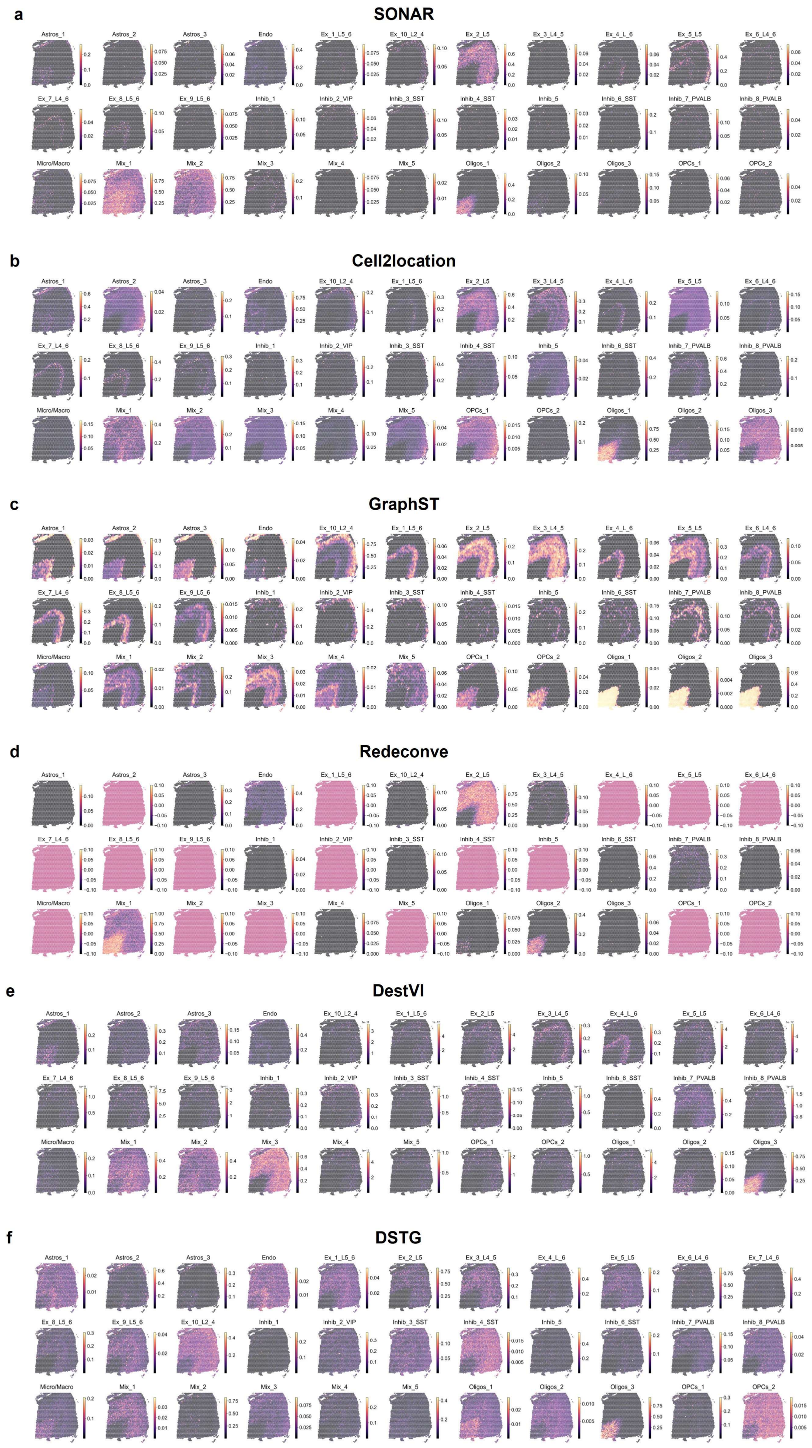

Figure S12. Deconvolution results of reference-based methods on the human dorsolateral prefrontal cortex dataset. **a** Deconvolved cell type proportions of SONAR. **b** Deconvolved cell type proportions of Cell2location. **c** Deconvolved cell type proportions of GraphST. **d** Deconvolved cell type proportions of Redeconve. **e** Deconvolved cell type proportions of DestVI. **f** Deconvolved cell type proportions of DSTG. The color of each spot represents the proportion of each cell type.

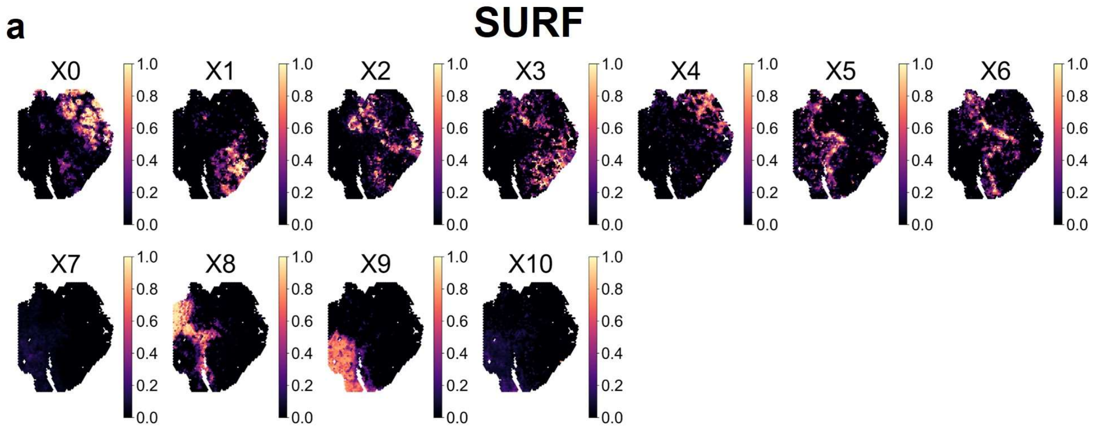

Figure S13. Analysis of the human colorectal liver metastasis dataset. a Deconvolved cell type proportions of SURF. The color of each spot represents the proportion of each cell type.

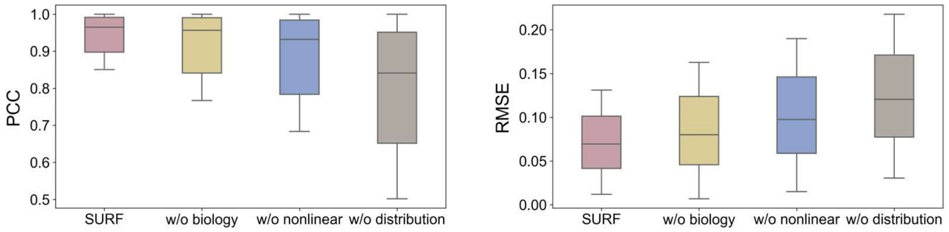

Figure S14. Ablation studies of SURF. Boxplots of Pearson correlation coefficient and root mean square error for the full SURF model and its ablated variants across all spots in six simulated datasets. The terms “w/o biology,” “w/o nonlinear,” and “w/o distribution” denote model variants where the strategies of incorporating biological prior knowledge, modeling nonlinear gene interactions, and applying the distribution regulation loss, respectively, have been removed. Center line, median value; box limits, upper and lower quartiles; whiskers, 0.5× interquartile range.

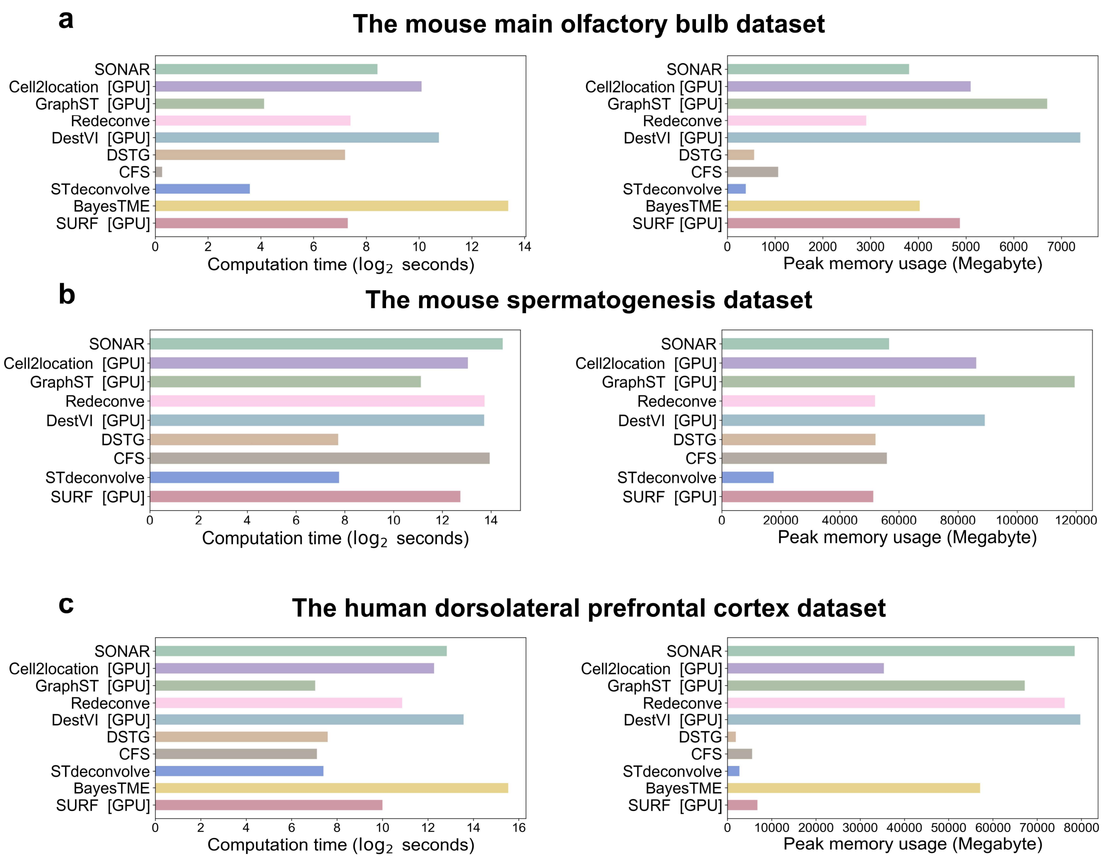

Figure S15. Benchmarking results of computational resources on a the mouse main olfactory bulb dataset, **b** the mouse spermatogenesis dataset, and **c** the human dorsolateral prefrontal cortex dataset. [GPU] indicates methods run on a graphics processing unit.

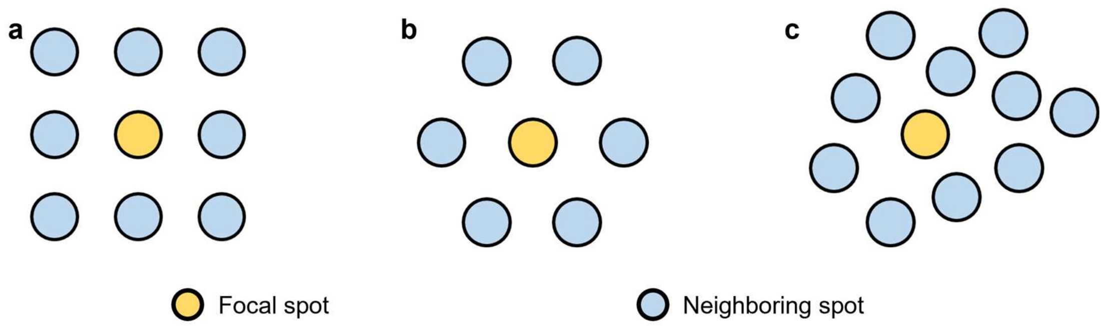

Figure S16. Definition of neighboring spots in different kinds of spatial modes. a Regular spatial mode in square shape. **b** Regular spatial mode in hexagonal shape. **c** Irregular spatial mode.

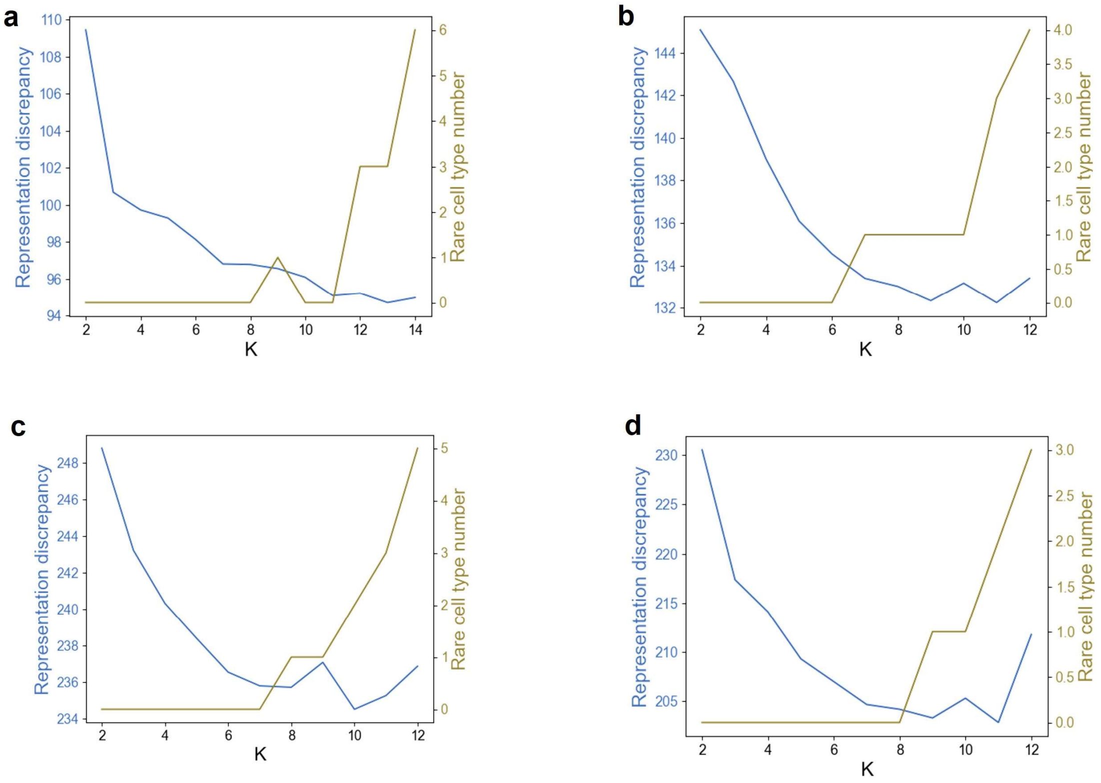

Figure S17. Selection of the number of cell types across different datasets. The plot of cell type number K versus representation discrepancy and rare cell type number in **a** the mouse main olfactory bulb dataset, **b** the mouse spermatogenesis dataset, **c** the human dorsolateral prefrontal cortex dataset, and **d** the human colorectal liver metastasis dataset.

Supplementary Tables

Table S1. Top ten upregulated genes of each cell type deconvolved by SURF in the mouse main olfactory bulb dataset.

	SURF									
	**Gene1**	**Gene2**	**Gene3**	**Gene4**	**Gene5**	**Gene6**	**Gene7**	**Gene8**	**Gene9**	**Gene10**
**X0**	Sox11	Mag	Tubb2b	Camk4	Cldn11	Nrgn	Necab2	Tyro3	Kcnh3	Gm13889
**X1**	Camk4	Necab2	Penk	Nrgn	Gpsm1	Fam131b	Cpne4	11-Mar	Icam5	Ubtf
**X2**	Ankrd29	Fam126a	AI848285	Ablim3	Sema3c	Gda	Stxbp6	Egr1	Rasl11b	Rbfox3
**X3**	Spp1	Shisa3	Reln	Uchl1	Elavl2	Sv2b	Slc17a7	Lhfpl3	Cacnb4	Gabrb2
**X4**	Doc2g	Cck	Cbln4	Slc17a7	Zfp804a	Mylk	Nptx1	Cdhr1	Lpgat1	Rab3b
**X5**	Mitf	Cbln4	Cck	Nptx1	Zfp804a	Cdhr1	Snrk	Kcna2	Plcxd2	Eomes
**X6**	Vip	Cdr1	Gap43	Camk2a	Ifi27l2a	Ptn	Shisa3	Doc2g	Lhfpl3	Scgb1c1
**X7**	Apold1	AI593442	Trh	Pde1c	Nxph1	Nrsn1	Vsnl1	Plxna4	Pik3r3	Calb2
**X8**	Plxna4	Cbln4	Pde1c	Mrpl27	Nxph1	Ifit1	Ptprd	Dnajc6	Igf1	Spp1
**X9**	Smtnl2	Omp	Plekha4	Unc13c	Agt	Atf5	Zdhhc2	Col12a1	Hmgcs2	Igfbp4
**X10**	Bpifb9a	Itih2	Bpifb9b	Dcn	Col1a1	Tfap2b	Foxd3	Mybpc1	Col1a2	Ogn

Table S2. Top ten upregulated genes of each cell type deconvolved by STdeconvolve in the mouse main olfactory bulb dataset.

	**STdeconvolve**									
	**Gene1**	**Gene2**	**Gene3**	**Gene4**	**Gene5**	**Gene6**	**Gene7**	**Gene8**	**Gene9**	**Gene10**
**X0**	Camk4	Nrgn	Sox11	Tyro3	Gm13889	Mag	Necab2	Sez6	Tubb2b	11-Mar
**X1**	Rbfox3	Kcnh3	AI848285	Rasl11b	Icam5	Gpsm1	Ncdn	Stxbp6	Grasp	Ubtf
**X2**	Sv2b	Slc17a7	Cdhr1	Elavl2	Mitf	Cacnb4	Spp1	Zfp804a	Gabrb2	Atf3
**X3**	Vip	Cdr1	Gap43	Camk2a	Sst	Doc2g	Psd	Zfp804a	Fam102b	Agap2
**X4**	Alas2	Hba.a2	Hbb.b2	Tfap2b	Mylk	Igf2	Ogn	Cp	X2410131<br>K14Rik	11-Mar
**X5**	Nxph1	Plxna4	AI593442	Apold1	Pde1c	Nrsn1	Pik3r3	Vsnl1	Trh	Calb2
**X6**	Kirrel	Smtnl2	Omp	Zdhhc2	Agt	Paqr6	Atf5	Unc13c	Plekha4	Hmgcs2
**X7**	Mybpc1	Fndc1	Npy	Ptn	Frzb	Sash1	Serpina3n	Vtn	Vat1l	Lmo4
**X8**	Bpifb9b	Gm14744	Bpifb9a	Bglap	Cyp2a5	Col1a1	Scgb1c1	Obp1a	Cd74	Tgtp1

Table S3. Top ten upregulated genes of each cell type deconvolved by CFS in the mouse main olfactory bulb dataset.

	CFS									
	**Gene1**	**Gene2**	**Gene3**	**Gene4**	**Gene5**	**Gene6**	**Gene7**	**Gene8**	**Gene9**	**Gene10**
**X0**	Nrgn	Mbp	Tubb2b	Camk4	Sox11	Cpne4	Sez6	Nrep	Tshz1	Camk2n1
**X1**	Pcp4	Camk2n1	Mbp	Hpcal4	Agap2	Camk2a	Camk2b	Penk	Gpsm1	Gng4
**X2**	Cdhr1	Slc17a7	Gabrb2	Gabra1	Sv2b	Reln	Shisa3	Chgb	Uchl1	Rab3b
**X3**	Cdr1	Map1b	Unc80	Map1a	Cacnb4	Slc1a2	Luzp2	Apc	Snhg11	Arap2
**X4**	Slc1a2	Doc2g	Slc6a11	X2900097C17Rik	Slc1a3	Cdr1	Map1a	Sparcl1	Map1b	Cck
**X5**	Ptgds	Apod	Beta.s	Fmod	Igf2	Bmp7	Kctd12	Alcam	Slc13a4	Ogn
**X6**	Nrsn1	Calb2	Nap1l5	Apold1	Vsnl1	Eef1a1	Th	AI593442	Pde5a	Frrs1l
**X7**	Nrsn1	Vsnl1	Pip5k1b	AI593442	Calb2	Nap1l5	Taf1	Shisa6	Fam178a	Shisa9
**X8**	Kctd12	Plp1	Apod	Npy	Sash1	Nr2f2	Frzb	Ptn	Ahcyl2	Fabp7
**X9**	Kif5b	Igfbp5	Apc	Atf5	Omp	Mcf2l	Nr2f2	Scd1	Hmgcs2	Plekhb1
**X10**	Omp	Apod	Fabp7	Scd1	Mcf2l	S100a5	Gng13	Hmgcs2	Igfbp5	Kctd12
**X11**	Hba.a2	Beta.s	Kalrn	Pcp4	Rasl11b	Meis2	Dixdc1	Ppp3r1	Mtfr1l	X4833424O15Rik
**X12**	Calm2	Irf1	Rps6	Fosl2	Dlx6as1	Col4a5	Pdzk1	Atp1a1	Fhl1	Postn
**X13**	Atp2b1	Arf3	Vamp2	Penk	Cck	Arhgef3	Necab2	Prkce	Bai2	Snapc1

Table S4. Top ten upregulated genes of each cell type deconvolved by BayesTME in the mouse main olfactory bulb dataset.

					BayesTME					
	**Gene1**	**Gene2**	**Gene3**	**Gene4**	**Gene5**	**Gene6**	**Gene7**	**Gene8**	**Gene9**	**Gene10**
**X0**	Nrgn	Rbfox3	Penk	Camk4	Icam5	Sez6	Cpne4	Gpsm1	Gng4	Mbp
**X1**	Stxbp6	Gad1	Ypel5	Ankrd29	Rasl11b	Pcp4	Mef2c	Sema3c	Pcp4l1	Atp1a1
**X2**	Pcp4	Rasl11b	Pcp4l1	Gad1	Arc	Stxbp6	Hpcal4	Ncdn	Inpp5j	4833424O15Rik
**X3**	Gad2	9330159F19Rik	Elmo1	Cox14	Caprin1	Vapa	Kifap3	Psmb5	Atp6v1g1	Ncor2
**X4**	Gad1	Dnajb6	Atp1b1	Pisd-ps1	Ppia	Ptpro	Cct7	Sp8	Mtfp1	Slc8a1
**X5**	Trf	Rab11b	Slc32a1	Clta	Fam20c	Slmap	Pisd-ps1	Abi2	Ppia	Anp32a
**X6**	Cdr1	Slc1a2	Doc2g	Map1a	Luzp2	Dio2	Pla2g7	Gm20594	Kcnj10	Atp1a2
**X7**	Cdhr1	Slc17a7	Reln	Gabrb2	Gabra1	Rab3b	Uchl1	Ptprd	Cacnb4	Sv2b
**X8**	Cacnb4	Uchl1	Reln	Gabrb2	Cplx1	Gabra1	Ptprd	Shisa3	Chgb	Erc2
**X9**	Aqp4	Ncan	Nrsn1	Shisa9	Chst2	Arhgap5	Msi2	Gria1	Mt2	Gfap
**X10**	Beta-s	Hba-a2	Hbb-b2	Gfap	Vsnl1	Nap1l5	Aqp4	Eomes	Hipk1	Gria1
**X11**	Cck	Cdhr1	Reln	Slc17a7	Map1b	Sv2b	Plcxd2	Dnajc6	Syt7	Ptprd
**X12**	H3f3b	Ptgds	Zic1	Zeb2	Marcksl1	Arih1	Marcks	Wdr6	Vsnl1	Dcaf7
**X13**	Nr2f2	Omp	Scd1	Kctd12	Apod	Hmgcs2	Atf5	S100a5	Frzb	Ccnd2

Table S5. Top ten upregulated genes of each cell type deconvolved by SURF in the mouse spermatogenesis dataset.

	SURF									
	**Gene1**	**Gene2**	**Gene3**	**Gene4**	**Gene5**	**Gene6**	**Gene7**	**Gene8**	**Gene9**	**Gene10**
**X0**	Tnp2	Cby3	Cstl1	Prm1	Klk1b8	Paqr5	Tex37	Prss51	Tnp1	Spata32
**X1**	Car2	Gm498	Reep6	Lelp1	4933411K16Rik	4933402P03Rik	Azin2	Prm2	Spata18	Fam71f2
**X2**	RP23-477L20.1	Dyrk4	Tmco5b	1700122O11Rik	Csrnp1	Slc36a3	Pex5l	Cd46	Jup	4930405D11Rik
**X3**	1700007K09Rik	Cacna2d1	1700067P10Rik	Tex36	4921501E09Rik	Kcne3	Ftmt	4931409K22Rik	1700011A15Rik	1700020L24Rik
**X4**	Pabpc6	Dnaic2	Rpa1	Cfap97	Flywch1	Dnah8	4932431P20Rik	Phf2	Slc2a3	Ccdc38
**X5**	mt-Rnr2	Malat1	Cyp11a1	Agt	Akr1cl	Ephx1	Cyp17a1	Gstm1	Meg3	Vcam1

Table S6. Top ten upregulated genes of each cell type deconvolved by STdeconvolve in the mouse spermatogenesis dataset.

STdeconvolve										
	**Gene1**	**Gene2**	**Gene3**	**Gene4**	**Gene5**	**Gene6**	**Gene7**	**Gene8**	**Gene9**	**Gene10**
**X0**	Car2	Gm498	Reep6	Lelp1	Prm2	Spata18	Ubqlnl	X4933411K16Rik	Fam71f2	Azin2
**X1**	Cby3	Paqr5	Prm1	Tnp2	Cstl1	Klk1b8	Gm11998	Noxred1	Prss51	Spata32
**X2**	Csrnp1	Pex5l	Dyrk4	X1700122O11Rik	Spata31	Slc36a3	Cd46	X4930571N24Rik	Hsfy2	X1700093K21Rik
**X3**	Hsd3b6	Meg3	Agt	Auts2	Lcn2	Akr1cl	Gstm1	Lix1l	Star	Vcam1

Table S7. Top ten upregulated genes of each cell type deconvolved by CFS in the mouse spermatogenesis dataset.

	CFS									
	**Gene1**	**Gene2**	**Gene3**	**Gene4**	**Gene5**	**Gene6**	**Gene7**	**Gene8**	**Gene9**	**Gene10**
**X0**	Prm1	Tnp2	Tnp1	Hmgb4	Fam71f1	Iqcf3	H1fnt	Gapdhs	Gsg1	Mlf1
**X1**	Rnf151	Tex33	Cypt12	Cypt4	Kif2b	Scp2d1	Fam71b	Hils1	Tulp2	Tuba8
**X2**	Spz1	Gapdhs	Fam71f2	Gsg1	Prm2	Spem1	Spata3	X4933411K16Rik	X1700027A15Rik	Reep6
**X3**	Odf1	Smcp	X4930571K23Rik	X4933411K16Rik	Reep6	Oaz3	Osbp2	Lelp1	Spata18	Azin2
**X4**	Lyar	Pabpc6	Nasp	X1700123L14Rik	Rsph1	Gkap1	Ccdc38	Dnah8	Dmrtb1	Phospho2
**X5**	Piwil1	Calm2	Ccp110	Lyar	Setx	Rsph1	Atxn7l3b	Ybx3	Mllt10	Pabpc1
**X6**	Clu	Calm1	mt.Nd1	Rsph1	Hspa5	Hsp90b1	Lyar	Sparc	mt.Nd4	Dazl
**X7**	Nasp	Sycp1	Pabpc1	Clu	Hsp90b1	Hspa5	Dazl	Hnrnpa2b1	Lyar	Calr
**X8**	Clu	mt.Nd1	Piwil1	Amhr2	Calm2	mt.Nd4	H3f3b	Tex29	Slfnl1	Cypt12
**X9**	mt.Rnr2	mt.Rnr1	mt.Nd1	X1700011A15Rik	Tsga8	Fam209	Tekt4	Tekt2	X1700001P01Rik	mt.Nd4
**X10**	Malat1	Ptgds	Gstm1	Agt	Aldh1a1	Cyp11a1	Cyp17a1	Lcn2	Akr1cl	Fabp3
**X11**	Camk1d	Hexb	Cmss1	Lars2	Gm15564	Mir6236	Gphn	Cdk8	Rn18s.rs5	X1700011A15Rik

Table S8. Top ten upregulated genes of each cell type deconvolved by SURF in the human dorsolateral prefrontal cortex dataset.

	SURF									
	**Gene1**	**Gene2**	**Gene3**	**Gene4**	**Gene5**	**Gene6**	**Gene7**	**Gene8**	**Gene9**	**Gene10**
**X0**	AQP1	MBP	BCAS1	GFAP	TP53INP2	PAQR6	FGF1	MOBP	AC005921.2	AIF1L
**X1**	GJB1	MOG	CLDN11	AL359091.1	ST18	KLK6	SLC5A11	PLP1	ABCA8	GLDN
**X2**	NR4A2	KRT17	SEMA3E	ISLR	TBR1	CPB1	DIRAS2	CLSTN2	PDE1A	ADRA2A
**X3**	PCP4	PCP4L1	FRMPD2	ALPL	RORB	SMYD2	ADRA1D	TOX	CXXC4	TMSB10
**X4**	SYT2	NEFH	NEFM	PLCH1	VAMP1	SV2C	RIT2	CTXN3	GPX3	NEFL
**X5**	PENK	C1QL2	LINC00507	CARTPT	HPCAL1	CALB1	LINC01007	LAMP5	PPP4R4	CALB2
**X6**	ACTA2	ADAMTS1	TPM2	MYL9	TAGLN	RELN	CALD1	MT1G	MALAT1	STON2
**X7**	NPY	HBA2	HBB	HBA1	CRHBP	CORT	SST	AC005944.1	PRR3	AC011603.3
**X8**	SCGB2A2	SCGB2A1	SCGB1D2	TFF1	AGR3	AGR2	PIP	MGP	COL1A1	UBN2
**X9**	IGHG3	IGKC	IGHM	IGHG4	IGLC2	IGHA1	IGHA2	PLIN1	G0S2	COL1A1

Table S9. Top ten upregulated genes of each cell type deconvolved by STdeconvolve in the human dorsolateral prefrontal cortex dataset.

	**STdeconvolve**									
	**Gene1**	**Gene2**	**Gene3**	**Gene4**	**Gene5**	**Gene6**	**Gene7**	**Gene8**	**Gene9**	**Gene10**
**X0**	HBA2	HBB	HBA1	ACTA2	MYL9	CPB1	ADAMTS1	TPM2	TAGLN	CALD1
**X1**	CUX2	C1QL2	CALB2	PENK	LINC00507	CARTPT	VIP	CALB1	HPCAL1	SAA2
**X2**	SLITRK4	ADCYAP1	NGEF	KCNC1	CACNG3	RARB	RNF215	TUBA4A	FGF9	CTXN3
**X3**	GAL	NEFH	PCSK1	NEFL	VAMP1	SYT2	NEFM	RIT2	FRMPD2	OXR1
**X4**	SCGB2A2	SCGB1D2	SCGB2A1	TFF1	AGR3	AGR2	COL1A1	PIP	MGP	CCND1
**X5**	ALPL	FSTL5	STMN2	UST	PDE1A	PLCH1	RPRM	PARM1	SNCB	SLC25A46
**X6**	MOG	GPIHBP1	GJB1	ERMN	AL359091.1	C21orf91	SLC5A11	MAG	HHIP	TMEM144
**X7**	NPY	IGHG3	IGLC2	IGKC	IGHG4	IGHM	CORT	IGHA1	CRHBP	IGHA2

Table S10. Top ten upregulated genes of each cell type deconvolved by CFS in the human dorsolateral prefrontal cortex dataset.

	CFS									
	**Gene1**	**Gene2**	**Gene3**	**Gene4**	**Gene5**	**Gene6**	**Gene7**	**Gene8**	**Gene9**	**Gene10**
**X0**	PLP1	CNP	CRYAB	TF	MBP	MAG	PPP1R14A	PTGDS	CLDND1	MOBP
**X1**	MBP	PLP1	GFAP	MOBP	CRYAB	CNP	S100B	FTH1	TF	BCAS1
**X2**	MT-CO1	MT-CO2	MT-CO3	MT-ND3	MT-ND1	MT-ND5	MT-ATP6	MTRNR2L12	MT-ND4	MT-ND2
**X3**	TMSB10	PCP4	SNAP25	TUBA1B	SYT1	TUBB2A	DIRAS2	STMN1	COX6C	SLC17A7
**X4**	NEFL	NEFM	NEFH	SNAP25	UCHL1	MAP1B	TUBA1B	VSNL1	SNCG	NRGN
**X5**	SCGB2A2	SCGB1D2	MGP	TFF1	MUC1	TFF3	KRT19	AZGP1	XBP1	MALAT1
**X6**	CST3	SLC1A2	CLU	APOE	AQP4	GJA1	AGT	ATP1B2	CPE	MT3
**X7**	ATP1B1	ACTB	NME7	ENC1	AC005944.1	LRRC75A	SYNC	AC011603.3	NORAD	SPTBN1
**X8**	SNAP25	GPM6A	CHN1	KRT17	SYT1	DIRAS2	CCK	SLC17A7	SCGB2A2	MT-CO1
**X9**	IGKC	IGLC2	IGHG3	IGHG4	IGHA1	IGHG1	IGHM	IGLC3	JCHAIN	IGHA2
**X10**	SST	GAD1	PVALB	TAC1	CXCL14	GAD2	GNAS	SLC6A1	SLC32A1	MALAT1
**X11**	HBB	HBA2	HBA1	ENC1	NRGN	MALAT1	CCK	GPM6A	CHN1	TMSB10

Table S11. Top ten upregulated genes of each cell type deconvolved by BayesTME in the human dorsolateral prefrontal cortex dataset.

	**BayesTME**									
	**Gene1**	**Gene2**	**Gene3**	**Gene4**	**Gene5**	**Gene6**	**Gene7**	**Gene8**	**Gene9**	**Gene10**
**X0**	CCK	HOPX	ENC1	MEF2C	NRXN1	LMO4	CAMK2A	C11orf87	NSG2	YWHAH
**X1**	HPCAL1	ENC1	SERPINE2	CAMK2N1	HOPX	MALAT1	ATP2B1	NPTXR	PPP3CA	CYP46A1
**X2**	PCP4	TMSB10	TUBB2A	NEFL	DCLK1	STMN2	PFKP	SLC24A2	TUBA1B	DIRAS2
**X3**	NEFM	NEFL	SNCG	SCN1B	VGF	CRYM	INA	CABP1	MDH1	NSF
**X4**	MT-ATP8	MTRNR2L8	MTRNR2L12	MT-ND2	MT-ND5	MT-ND4L	MT-ND6	MT-ND1	MT-ATP6	MT-ND3
**X5**	PLP1	MOBP	MAG	TF	ERMN	PPP1R14A	CLDN11	CNP	MBP	SPP1
**X6**	HBA2	HBB	APOE	GJA1	SLC1A2	ATP1A2	CST3	ATP1B2	AQP4	SLC1A3
**X7**	MT-ND4L	MT-ATP8	MT-CO3	MT-CO2	NCS1	MT-ND3	MTRNR2L12	MT-CO1	MT-CYB	MTRNR2L8
**X8**	SCGB2A2	SCGB1D2	NPY	IGKC	MUC1	KRT19	SST	MGP	HLA-B	HLA-A
**X9**	RTN1	CABP1	ATP1A1	DLGAP1	ANXA6	PPP3R1	NPTN	VSNL1	NELL2	MDH1
**X10**	DIRAS2	NAP1L3	STMN1	SNCA	PGM2L1	PHYHIPL	G3BP2	TSPYL4	TCEAL7	LY6H
**X11**	ATP1B1	YWHAH	VSNL1	NPTN	RGS4	SCG5	CHN1	RBFOX1	C11orf87	PPP3R1

Table S12. Top ten upregulated genes of each cell type deconvolved by SURF in the human colorectal liver metastasis dataset.

	**SURF**									
	**Gene1**	**Gene2**	**Gene3**	**Gene4**	**Gene5**	**Gene6**	**Gene7**	**Gene8**	**Gene9**	**Gene10**
**X0**	FAM3B	RPS12	RPS27A	RPL22	AREG	SNHG5	MAGOH	SNRPD1	NDUFB3	TATDN1
**X1**	CKB	SLC6A8	SLC2A1	PIGZ	NDRG1	ANKRD37	EFNA1	MTMR11	FAM13A	VEGFA
**X2**	AC078993.1	AC015813.1	AMY2B	PABPC1L	UCA1	DIO3OS	KIFC2	HOXB6	NR4A1	HOXB9
**X3**	FNDC1	COL8A1	NDUFA4L2	COL4A1	COL4A2	DKK3	CNN1	VCAN	IGFBP5	COL1A1
**X4**	OGN	FRZB	SLIT3	CLDN5	FOXS1	ELN	MUC5B	CSPG4	WFDC2	INMT
**X5**	IGHA1	IGKC	JCHAIN	IGHG4	IGHM	PTGDS	IGLC3	IGHG1	IL7R	IGHG2
**X6**	MMP9	SLC11A1	SLC15A3	TREM2	LILRB4	CCL18	ITGB2	ITGAX	ACP5	IFI30
**X7**	PLA2G2A	SERPINA7	SERPINA1	FGL1	ORM1	HAO1	HPX	FGG	OGDHL	AC008760.2
**X8**	CYP2A6	ALB	CYP1A2	GSTA1	GNMT	UGT2B7	PLGLB2	TTR	SERPINA4	SLC22A1
**X9**	PLA2G2A	SAA2	FGL1	SAA1	AC008760.2	CPN1	CRP	ORM1	HAMP	SERPINA1
**X10**	CRYAB	SAA1	UNC5B	HAMP	SAA2	APOA4	MT1H	BAAT	MT1M	CFHR5

Supplementary Methods

Spot simulation

For individual spot simulation in the fisrt group of ST data, we first sampled from the Conway–Maxwell Poisson distribution (default expectation 12) at random to determine the total cell number per spot. Then we applied Dirichlet distribution to determine the proportions of six cell types within a spot, the alpha of Dirichlet distribution is set as 0.2 to model the sparsity of cell type distribution. We selected the highest proportion values to represent the dominating type. The other five proportion values were randomly allocated to remaining five cell types. Subsequently, based on the previously determined total cell number and cell type proportions (CTPs), we calculated the cell number of each cell type within a spot. Finally, we sampled cells from the annotated single-cell dataset according to the calculated cell number for each cell type. And the gene counts of all cells were then accumulated to obtain the gene profile of each spot. Through the above steps, we created a simulated ST spot with gene expressions and ground truth CTPs.

For individual spot simulation in the second group of ST data, we also first sampled from the Conway–Maxwell Poisson distribution (default expectation 12) at random to determine the total cell number per spot. Then we applied Dirichlet distribution to determine the proportions of eight cell types within a spot, the alpha of Dirichlet distribution is set as 0.2 to model the sparsity of cell type distribution. For each spot, we randomly selected one of the two highest proportion values to designate the dominant cell type. The lowest three proportion values were reassigned, at random, to match the dominant cell types from the other four regions. The remaining three proportion values were randomly allocated to the three dispersed cell types. Subsequently, based on the previously determined total cell number and CTPs, we calculated the cell number of each cell type within a spot. Finally, we sampled cells from the annotated single-cell dataset according to the calculated cell number for each cell type. And the gene counts of all cells were then accumulated to obtain the gene profile of each spot. Through the above steps, we created a simulated ST spot with gene expressions and ground truth CTPs.

Deconvolution of scRNA-seq simulated ST data

We conducted the default preprocessing steps for simulated ST datasets. We trained SURF and other reference-free methods using $K = 6$ for the first group of simulated data and $K = 8$ for the second group of simulated data. To match the deconvolved cell types with the true cell types for all reference-free data, we calculated the Pearson correlation coefficients between the deconvolved and the true cell type transcriptional profiles. Each deconvolved cell type is assigned to the true cell type with the highest Pearson correlation value.

In the evaluation of reference-based methods, we considered three scenarios of scRNA-

seq reference data: ideal reference, cell type missing reference, and real reference. Ideal reference is the original scRNA-seq data that used to simulate ST data. Cell type missing reference is produced by dropping out acinar cell type from the ideal reference. And the real reference is an annotated human pancreas scRNA-seq dataset[1] from another human sample in the same study. In this scRNA-seq dataset , we chose the cell types that present in the simulated ST data, yielding a reference comprising six cell types consistent with the simulated ST data.

Deconvolution of mouse main olfactory bulb data

The mouse main olfactory bulb (MOB) data contained measured counts of 16,034 genes for 282 spots.[1] After the default preprocessing process, we trained SURF using a range of K from 2 to 14 and selected $K = 11$, which produced the lowest representation discrepancy when the number of rare cell types was 0 (**Figure S17a**). To identify the upregulated genes in the deconvolved transcriptional profile of the rostral migratory stream (RMS)-related cell type, we calculated the $\mathrm{Log}_2\,\mathrm{fold\_change}$ of the corresponding deconvolved transcriptional profile with respect to the mean deconvolved expression of the other deconvolved cell types.

Deconvolution of mouse spermatogenesis data

There are counts of 24,450 genes for 31,659 spots in the mouse spermatogenesis data.[2] Considering the small number of cells per spot in the high-resolution data, we excluded only those spots with 50 or fewer gene counts. Then we removed genes present in all the spots or detected in fewer than 100 spots. Next, we selected the top 500 significantly overdispersed genes and scaled the total gene counts of each spot to 1. The same preprocessing standards was also applied for STdeconvolve except the scaling. After preprocessing, we trained SURF using a range of K from 2 to 12 and selected the model $K = 6$, which produced the lowest representation discrepancy when the number of rare cell types was 0 (**Figure S17b**). The batch size was set as 256 to accelerate computation.

Deconvolution of human dorsolateral prefrontal cortex data

The raw count matrix of the human dorsolateral prefrontal cortex data included 33,538 genes for 3,639 spots.[3] After the default preprocessing process, we fitted the SURF model with a range of K from 2 to 12 and chose the model $K = 10$, which achieved the lowest representation discrepancy in the entire range (**Figure S17c**).

Deconvolution of human colorectal liver metastasis data

The human colorectal liver metastasis data included 1,537 spots with 36,601 measured genes.[4] We implemented the default preprocessing process first, and searched for the best SURF model in the range of K from 2 to 12. We selected the model at $K = 11$,

which produced the lowest representation discrepancy in the entire range (**Figure S17d**). In the analysis of this dataset, we calculated epithelial-to-mesenchymal transition (EMT) scores for spots in the cancer region. To calculate EMT scores, we first used the 'sc.pp.normalize_total' function to normalize ST data. Then we used the 'sc.tl.score_genes' function to calculate EMT scores for each spot, and the 'gene_list' in the function was set as the EMT-related genes listed in the EMTome database.[5] To explore the concordant differences between cell type X0 (epithelial state) and X1 (mesenchymal state), we conducted hallmark gene set enrichment analysis (GSEA). We calculated the Log_2 fold_change of the deconvolved transcriptional profile of X1 with respect to the deconvolved transcriptional profile of X0, and the Log_2 fold_change results were sorted in descending order and used as input for GSEA analysis. The GSEA analysis was performed using the 'clusterProfiler' package.

Evaluations in simulation data

In the simulation data, we calculated Pearson correlation coefficient (PCC) and root mean square error (RMSE) of each spot to compare different methods, which are defined as:

$$\text{PCC} = \frac{E[(\boldsymbol{\theta}_s^{pred} - \mathrm{E}(\boldsymbol{\theta}_s^{pred}))(\boldsymbol{\theta}_s^{true} - \mathrm{E}(\boldsymbol{\theta}_s^{true}))]}{\sigma_{\boldsymbol{\theta}_s^{pred}} * \sigma_{\boldsymbol{\theta}_s^{true}}}$$

$$\text{RMSE} = \sqrt{\frac{\sum_{k=1}^{K}(\theta_{sk}^{pred} - \theta_{sk}^{true})^2}{K}}$$

where K is the number of cell types, $\boldsymbol{\theta}_s^{pred} = \{\theta_{s1}^{pred}, \theta_{s2}^{pred}, \dots, \theta_{sK}^{pred}\}$ is the predicted CTPs for spot s, and $\boldsymbol{\theta}_s^{true} = \{\theta_{s1}^{true}, \theta_{s2}^{true}, \dots, \theta_{sK}^{true}\}$ is the ground truth CTPs for spot s**.** The higher PCC and the lower RMSE mean the better deconvolution performance.

Evaluations in real data

In the MOB dataset, we calculated mean expression of RMS marker genes to compare different deconvolution algorithms. We first normalized the raw count matrix:

$$d_{sg}{}' = \frac{d_{sg}}{\sum_{g=1}^{G} d_{sg}}$$

$$d_{sg}{}'' = \frac{d_{sg}{}' - \min_s d_{sg}{}'}{\max_s d_{sg}{}' - \min_s d_{sg}{}'}$$

Then we calculated the mean expression of RMS's marker genes using the normalized count matrix:

$$\text{mean\_expr} = \frac{\sum_{s=1}^{S} d_{sg_m}{}''\theta_{sk_t}}{\sum_{s=1}^{S} \theta_{sk_t}}$$

where d_{sg} is the gene count of gene g at spot s. Gene g_m is the marker gene. θ_{sk_t} is the predicted cell type proportions of cell type k_t at the spots s, and cell type k_t is the deconvolved cell type corresponding to RMS.

In the mouse spermatogenesis dataset and human dorsolateral prefrontal cortex dataset, we used adjusted rand index (ARI) to access the deconvolution performance of different methods. The deconvolved cell type with the highest cell type proportions is used as the spot-level dominant cell type prediction of each spot. Then we calculated ARI based on the spot-level dominant cell type predictions and spot-level annotations. To calculate ARI, we first calculate a contingency table. Given a set of S spots, predicted groupings $\boldsymbol{X} = \{\boldsymbol{X_1}, \boldsymbol{X_2}, \dots, \boldsymbol{X_p}\}$ and ground truth groupings $\boldsymbol{Y} = \{\boldsymbol{Y_1}, \boldsymbol{Y_2}, \dots, \boldsymbol{Y_q}\}$. p and q are the numbers of subsets in predicted groupings and ground truth groupings respectively. A contingency table can summarize the overlap between $\boldsymbol{X}$ and $\boldsymbol{Y}$, whose definition is

$\boldsymbol{X}\backslash\boldsymbol{Y}$	$\boldsymbol{Y_1}$	$\boldsymbol{Y_2}$	...	$\boldsymbol{Y_q}$	sums
$\boldsymbol{X_1}$	n_{11}	n_{12}	...	n_{1s}	a_1
$\boldsymbol{X_2}$	n_{21}	n_{22}	...	n_{2s}	a_2
$\vdots$	$\vdots$	$\vdots$	$\ddots$	$\vdots$	$\vdots$
$\boldsymbol{X_p}$	n_{s1}	n_{s2}	...	n_{ss}	a_p
sums	b_1	b_2	...	b_q	

where n_{ij} is the number of spots in the intersection of $\boldsymbol{X_i}$ and $\boldsymbol{Y_j}$. Based on the contingency table, ARI is defined as:

$$\text{ARI} = \frac{\sum_{ij}\binom{n_{ij}}{2} - \left[\sum_i\binom{a_i}{2}\sum_j\binom{b_j}{2}\right]/\binom{S}{2}}{\frac{1}{2}\left[\sum_i\binom{a_i}{2} + \sum_j\binom{b_j}{2}\right] - \left[\sum_i\binom{a_i}{2}\sum_j\binom{b_j}{2}\right]/\binom{S}{2}}$$

References

[1] P. L. Ståhl *et al.*, “Visualization and analysis of gene expression in tissue sections by spatial transcriptomics,” *Science*, vol. 353, no. 6294, pp. 78–82, Jul. 2016.

[2] H. Chen *et al.*, “Dissecting mammalian spermatogenesis using spatial transcriptomics,” *Cell Rep.*, vol. 37, no. 5, p. 109915, Nov. 2021, doi: 10.1016/j.celrep.2021.109915.

[3] K. R. Maynard *et al.*, “Transcriptome-scale spatial gene expression in the human dorsolateral prefrontal cortex,” *Nat. Neurosci.*, vol. 24, no. 3, pp. 425–436, Mar. 2021, doi: 10.1038/s41593-020-00787-0.

[4] O. Garbarino *et al.*, “Spatial resolution of cellular senescence dynamics in human colorectal liver metastasis,” *Aging Cell*, vol. 22, no. 7, p. e13853, Jul. 2023, doi: 10.1111/acel.13853.

[5] S. V. Vasaikar *et al.*, “EMTome: a resource for pan-cancer analysis of epithelial-mesenchymal transition genes and signatures,” *Br. J. Cancer*, vol. 124, no. 1, pp. 259–269, Jan. 2021, doi: 10.1038/s41416-020-01178-9.
